# Dendritic cell‐derived lncRNAs in patients with acute coronary syndrome

**DOI:** 10.1111/jcmm.70057

**Published:** 2024-10-21

**Authors:** Zhenglong Wang, K. E. Changhao, Yuheng Chen, Yongchao Zhao, Yuanjie He, Xiao Liang, Qingxian Tu, Min Xu, Fujia Guo, Junbo Ge, Bei Shi

**Affiliations:** ^1^ Department of Cardiology The Third Affiliated Hospital of Zunyi Medical University Zunyi China; ^2^ Department of Cardiology Affiliated Hospital of Zunyi Medical University Zunyi China; ^3^ Department of Cardiology, Shanghai Institute of Cardiovascular Diseases, Zhongshan Hospital Fudan University Shanghai China

**Keywords:** acute coronary syndrome, gene sequencing, long non‐coding RNA, monocyte‐derived dendritic cells, pathogenesis

## Abstract

Long non‐coding RNAs (lncRNAs) and dendritic cells (DC) play crucial roles in the development of acute coronary syndrome (ACS); however, the mechanisms remain unclear. To investigate this, we analysed the differentially expressed lncRNAs in monocyte‐derived DCs (moDCs) from patients with ACS. Peripheral blood mononuclear cells were transformed into moDCs. Cellular morphology and expression levels of moDC‐specific markers (CD80, CD86, CD11c, CD14 and HLA‐DR) were analysed using electron microscopy (EM) and flow cytometry (FCM), respectively. Differentially expressed lncRNAs and their functions were predicted using gene sequencing, gene ontology and the Kyoto Encyclopedia of Genes and Genomes. The expression levels of markers, signalling pathway molecules (p‐PI3K and p‐AKT), inflammatory cytokines (IL‐6 and IL‐12p70) and target gene (C‐C motif chemokine ligand (*CCL*) 15 and *CCL14*) were analysed by overexpression or silencing of candidate lncRNAs. EM revealed the cells to be suspended in dendritic pseudopodia. CD11c and HLA‐DR were upregulated, while CD80 and CD86 were downregulated. Comparison between the UA versus ST group showed the highest number of differentially expressed lncRNAs (*n* = 113), followed by UA versus NST (*n* = 115), CON versus NST (*n* = 49) and CON versus ST (*n* = 35); however, the number was low for CON versus UA and ST versus NST groups. moDC‐specific marker expression, signalling pathway molecules, inflammatory cytokines and *CCL14* were upregulated following lentiviral overexpression of smart silencer‐*CCL15‐CCL14*; however, expression levels decreased following transfection with siRNA. The morphology, function and lncRNA expression of moDCs differ depending on the type of ACS. The differentially expressed lncRNAs, particularly CCL15‐CCL14, regulate the function of moDCs. Thus, our study provides new insights regarding the role of lncRNAs in ACS and indicates the potential use of CCL15‐CCL14 as a novel diagnostic marker and therapeutic target.

## 1 INTRODUCTION

Acute coronary syndrome (ACS) is a global health concern involving unstable angina and myocardial infarction due to sudden blood flow blockage to the coronary artery. Several studies have focused on understanding the pathogenesis of ACS, with a particular emphasis on local mechanisms related to plaque characteristics and environmental factors.^1^, ^2^, ^3^ However, Ounzain et al.^4^ characterized cardiac long non‐coding RNAs (lncRNAs) post‐myocardial infarction and identified hundreds of novel heart‐specific lncRNAs with unique regulatory and functional characteristics and reported that cardiac disease results from the dysregulation of gene regulatory networks. Hence, the regulatory factors of gene expression, rather than local and environmental factors, are of great interest in understanding the pathogenesis and progression of ACS plaques.^5^ Gene expression regulation plays a critical role in determining the characteristics of atherosclerotic plaques, including their stability and vulnerability to rupture.

LncRNAs were once considered the “dark matter” and “noise” of genome because they were poorly understood and thought to lack biological functions. They were initially discovered as products of RNA transcription but were not believed to have a significant role in gene regulation or other cellular processes.^6^ However, research over the past few decades has implicated them in various diseases, including the occurrence and development of ACS.^7^, ^8^, ^9^ Additionally, research has suggested that immunity plays a crucial role in the development and progression of atherosclerosis, the underlying cause of ACS.^10^, ^11^ Based on the above findings, we hypothesized the lncRNAs of monocyte‐derived dendritic cells (moDCs) to be associated with the onset and progression of ACS.

This study aimed to determine the role and mechanism of DC‐derived lncRNA in patients with ACS, to provide a new perspective and reference for further exploration of ACS pathogenesis, which may be important for identifying potential therapeutic targets and developing strategies to prevent or treat ACS.

## 2 METHODS

### 2.1 Study cohort

In the present study, patients with ACS were divided into four groups: unstable angina (UA), non‐ST‐segment elevation myocardial infarction (NSTEMI or NST), ST‐segment elevation myocardial infarction (STEMI or ST) and normal (control, CON). The protocol was approved by the local ethics committee and all patients signed an informed consent form.

### 2.2 moDC culture and identification

Peripheral blood mononuclear cells (PBMC) were isolated, drawn from the patients, using Ficoll density gradient centrifugation and collected using an immunomagnetic bead isolation column (MACS) containing CD14 antibodies (Miltenyi, Germany). Recombinant rat granulocyte‐macrophage colony‐stimulating factor (GM‐CSF) (20 ng/mL) (PeproTech, USA) and recombinant human (rh) interleukin (IL)‐4 (20 ng/mL) (PeproTech, USA) were added to PBMCs and incubated at 37°C to induce the transformation into moDCs.

The moDCs were identified based on their morphologies using an inverted phase‐contrast microscopy (OLYMPU, Germany) and scanning and transmission electron microscopy (SEM, TEM), (HITACHI, Japan), respectively. The expression levels of the moDC markers CD80, CD86 and HLA‐DR (Biolegend, USA)were detected using flow cytometry (FCM).

### 2.3 Morphological and functional differences among moDCs

Morphological differences in moDCs among patients with different types of ACS were compared using SEM. The expression levels of moDC markers CD80, HLA‐DR and ovalbumin (OVA) were detected using FCM to investigate the ability of antigen presentation and phagocytosis. Additionally, the expression levels of IL‐6, IL10 and IL12p70 were detected using enzyme‐linked immunosorbent assay (ELISA) (Beijing Solarbio, China) to evaluate the secretion capability.

### 2.4 Differences in lncRNA expression

Differences in lncRNA expression in moDCs were analysed using the high‐throughput sequencing platform, Illumina HiSeq 3000 platform (RiboBio Co., Ltd., China), commonly used for RNA sequencing (RNA‐seq) studies, including the analysis of lncRNA expression. Differentially expressed lncRNAs were screened at a significance level (*q*‐value <0.05) and log2 fold change of >1. The accuracy of the differentially expressed lncRNAs was verified using reverse‐transcriptase polymerase chain reaction (RT‐PCR), and their biological functions were predicted using gene ontology (GO) and Kyoto Encyclopedia of Genes and Genomes (KEGG) analyses.

### 2.5 Roles and mechanisms of candidate lncRNAs

Functional changes in moDCs such as antigen presentation, cytokine secretion and the phosphorylation level of the moDC PI3K‐AKT protein pathway were evaluated using FCM, RT‐PCR and western blotting (WB) after overexpression and silencing of candidate lncRNAs in moDCs. The sub‐localization of candidate lncRNAs in moDCs was explored by fluorescence in situ hybridization, and their regulatory effect on the target gene was assessed using FCM, RT‐PCR and WB.

### 2.6 Statistical analyses

SPSS29.0 statistical software (IBM Corp., Armonk, NY, USA, Version 29.0) was used for all statistical analyses. The data were normally distributed and are expressed as the mean ± SD. Mann–Whitney *U* test was used to compare continuous variables between the two groups. Two test was used to compare qualitative data. The Holm–Sidak method was used for comparison among multiple groups. Linear regression and Spearman's rank correlation were used to evaluate the correlation between lncRNA levels and continuous variables, and logistic regression was used to evaluate the correlation between lncRNA levels and binary classification variables. *p* < 0.05 was considered as statistically significant difference.^12^

## 3 RESULTS

### 3.1 Characteristics of the study population

Characteristics of the study population are listed in Table 1.

**TABLE 1:** Study population characteristics.

Variable	Control (CON)	STEMI (ST)	NSTEMI (NST)	UA
	(*n* = 8)	(*n* = 9)	(n = 8)	(*n* = 6)
Age, years	45.67 ± 0.58	63.33 ± 19.55	57.33 ± 17.21	66.67 ± 5.69
SBP, mmHg	117 ± 6.25	142.33 ± 15.63	146.67 ± 30.55	127.33 ± 23.59
DBP, mmHg	76.33 ± 3.74	78.33 ± 7.64	84.33 ± 21.22	81.33 ± 5.69
HR, beats/min	77 ± 9.17	77 ± 8.54	73.33 ± 15.50	84 ± 13.53
WBC, ×109/L	8.45 ± 4.40	9.62 ± 3.47	9.78 ± 3.74	8.7 ± 1.58
TC, mmol/L	5.94 ± 0.61	4.44 ± 1.06*	4.63 ± 0.47*	3.75 ± 0.55*
TG, mmol/L	2.68 ± 1.16	2.17 ± 0.94	3.42 ± 2.10	1.22 ± 0.32
LDL‐C, mmol/L	3.60 ± 0.21	2.83 ± 0.78*	2.77 ± 0.23*	2.35 ± 0.58*
HDL‐C, mmol/L	1.39 ± 0.31	1.04 + 0.10*	1.03 + 0.15*	1.09 + 0.29*
CK, U/L	55.67 ± 2.52	112.33 ± 29.26	623.67 ± 478.73	51.67 ± 23.80
CK‐MB, U/L	19.67 + 1436	21 ± 1.0	44 + 3027	11.67 ± 1.53
TNT‐hs, ng/L	0.12 ± 0.03	2799.95 ± 4797.84	927.75 ± 782.42	1.21 + 186
Lipoprotein A, nmg/L	108.02 ± 59.56	212.41 ± 283.52	71.2 ± 74.51	88.27 ± 67.47
BNP, pg/ml	51.11 ± 21.58	2432.44 ± 2209.07	623.03 ± 656.14	61.91 ± 5.47

Abbreviations: CK, creatine kinase; CK‐MB, creatine kinase‐MB; CON, control, healthy volunteers; DBP, diastolic blood pressure; HDL‐C, high‐density lipoprotein cholesterol; HR, heart rate; hs‐TNT, troponin T (TnT); LDL‐C, low‐density lipoprotein cholesterol; NSTEMI, non‐ST‐segment elevation myocardial infarction; SBP, systolic blood pressure; STEMI, ST‐segment elevation myocardial infarction; TC, total cholesterol; TG, triglycerides; WBC, white blood cell.

* *p* < 0.05, compared to CON.

### 3.2 Identification of moDCs

**FIGURE 1:**
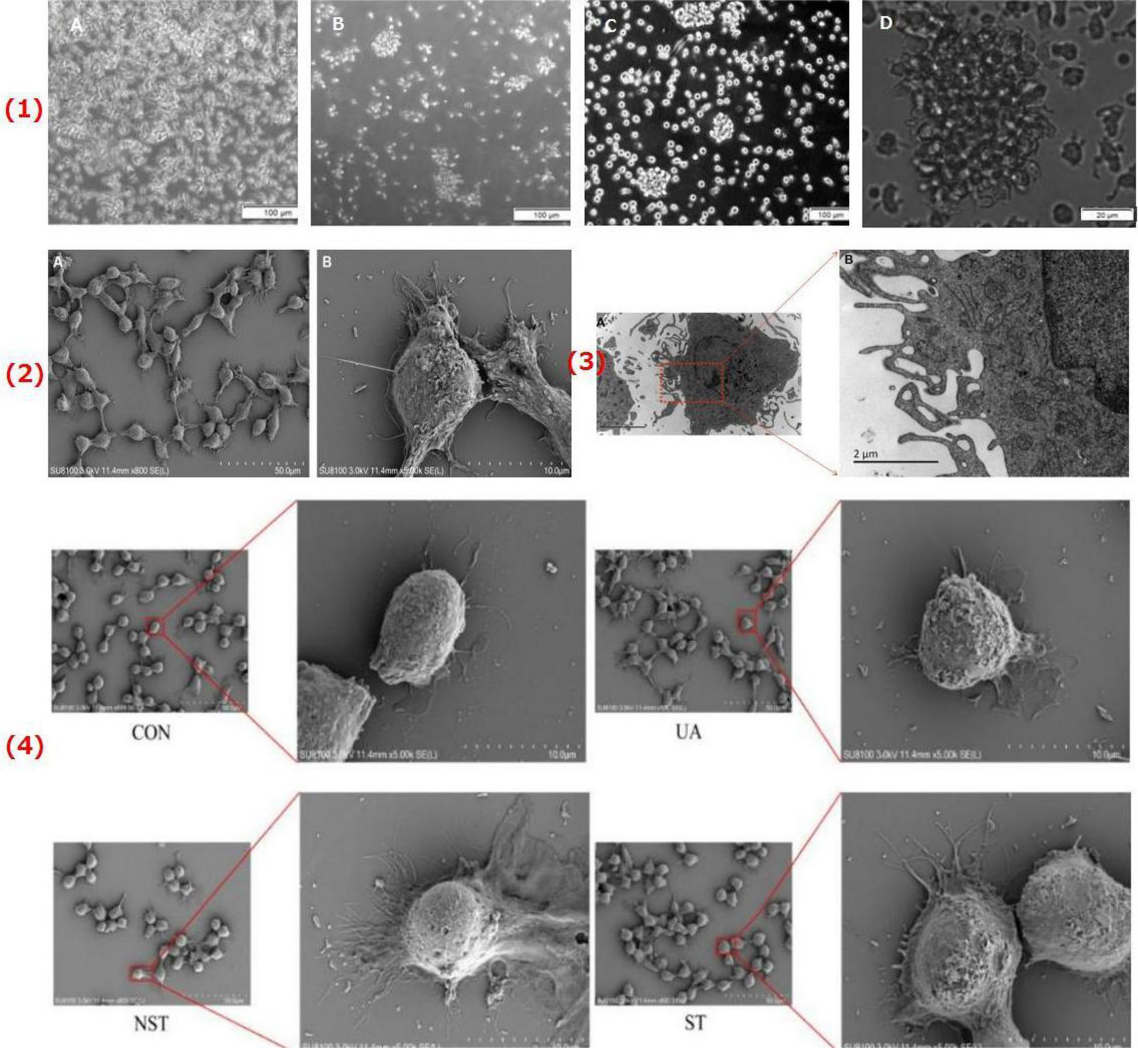
The morphology of moDC. (1) The moDC morphology observed with an inverted microscope. (A) Day 2 after MACS culture; (B) Day 4 after MACS culture; (C, D) Day 6 after MACS culture; (A, B, C) Scale bar = 100 μm; (D) Scale bar = 20 μm.MACS, magnetic‐activated cell sorting. (2) The moDC morphology observed with scanning electron microscope; (A) Scale bar = 50 μm; (B) Scale bar = 10 μm. (3) The moDC morphology observed with transmission electron microscope; (A) Scale bar = 5μm; (B) Scale bar = 2μm. (4) The morphological differences of moDCs derived from different groups as observed using scanning electron microscopy. moDCs, monocyte‐derived dendritic cells; UA, unstable angina; NST, non‐ST‐segment elevation myocardial infarction; ST, ST‐segment elevation myocardial infarction; CON, normal control.

**FIGURE 2:**
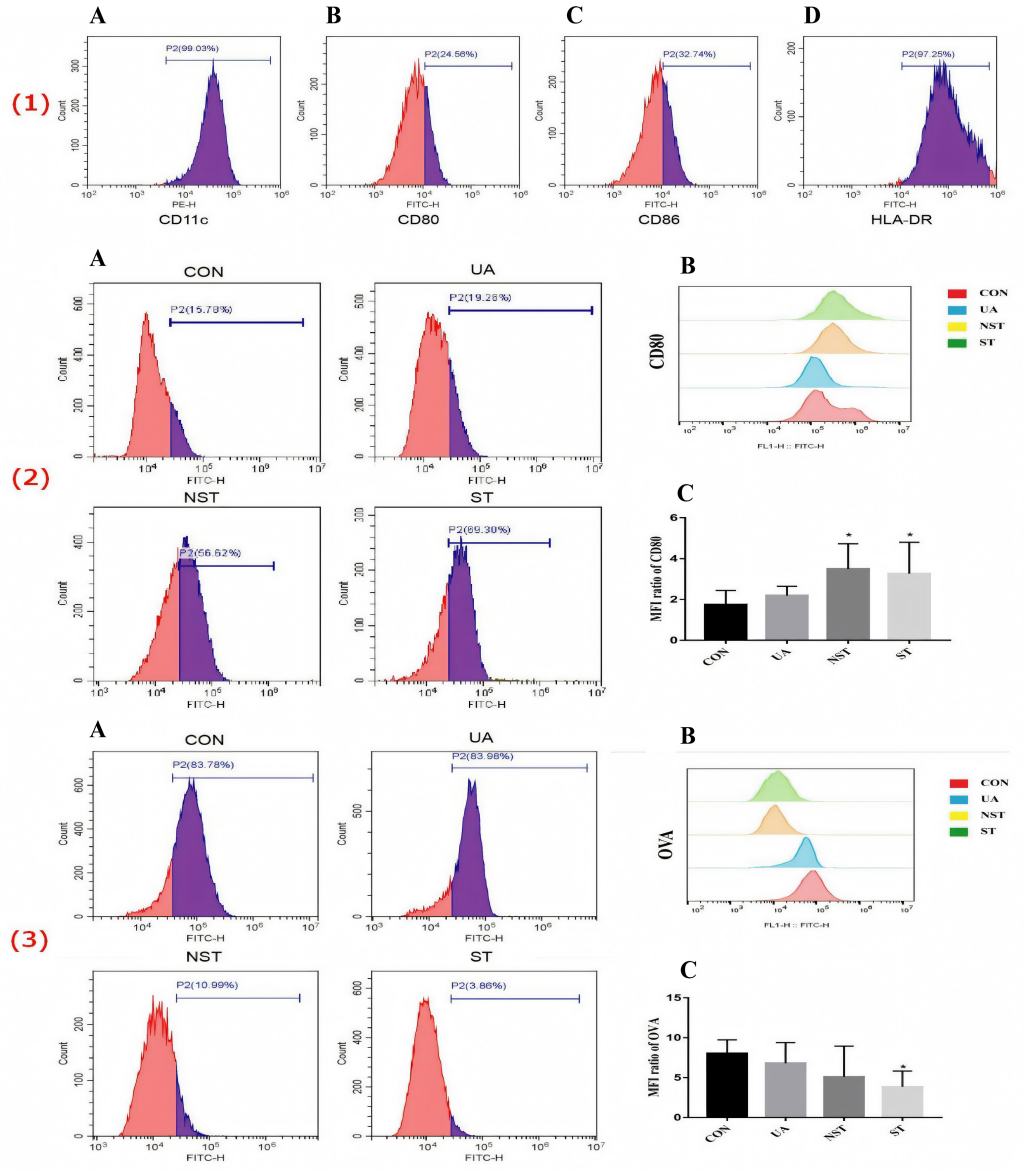
FCM results of markers expressed by the moDCs. (1) FCM results of markers expressed by normal moDCs. (A) The levels of CD11c were 99.02 ± 0.12%; (B) The levels of HLA‐DR were 99.32 ± 0.08%; (C) The levels of CD80 were 24.27 ± 0.99%; (D) The levels of CD86 were 32.38 ± 0.59%. (2) The differences in the antigen presentation capability of moDCs among different groups analysed using flow cytometry. (A, B) The expression levels of CD80 were 15.78, 19.28, 56.62 and 69.30% in the CON, UA, NST and ST groups, respectively. (C) The mean fluorescence intensity (MFI) values of CD80 among different groups. (3) The differences in the phagocytotic ability of moDCs among different groups analysed using flow cytometry. (A, B) The expression levels of OVA were 83.78, 83.98, 10.99 and 3.86% in the CON, UA, NST and ST groups, respectively. (C) The mean fluorescence intensity (MFI) values of OVA among different groups. moDCs, monocyte‐derived dendritic cells; FCM, flow cytometry; OVA, ovalbumin; UA, unstable angina; NST, non‐ST‐segment elevation myocardial infarction; ST, ST‐segment elevation myocardial infarction; CON, normal control.

Inverted phase‐contrast microscopy showed that on the Day 2 of culture, moDCs proliferated in a semi‐suspended and semi‐adherent form [Figure 1(1)A]. On Day 4, the cells grew as suspensions with irregular shapes and convex surfaces [Figure 1(1)B]; whereas on Day 6, the moDCs showed duckweed aggregation, large volume, obvious dendritic protrusions and a large number of colonies [Figure 1(1)C,D]. SEM showed that the moDCs had typical DC characteristics: an elliptical shape, rough surface and laminated folds [Figure 1(2)]. TEM showed that the moDCs were irregular in shape, with many protrusions of varying lengths and thicknesses at the edges, a large number of endoplasmic reticula and mitochondria, few lysosomes in the cytoplasm and an irregular nucleus [Figure 1(3)]. The results of FCM demonstrated that the levels of CD11c and HLA‐DR were high (99.02 ± 0.12% and 99.32 ± 0.08%, respectively), whereas those of CD80 and CD86 were low (24.27 ± 0.99% and 32.38 ± 0.59%, respectively) [Figure 2(1)].

### 3.3 Morphological and functional differences in moDCs among the study groups

Regarding the differences in morphology, the SEM images demonstrated that the moDCs of the UA, NST and ST groups had longer and denser pseudopod‐like processes than those of the CON group [Figure 1(4)].

The results of FCM showed that the expression levels of CD80 and HLA‐DR and mean fluorescence intensity (MFI) in the ST and NST groups were higher than those in the CON and UA groups (*p* < 0.05). However, there was no statistical difference in CD80 and HLA‐DR expression levels and antigen presentation by moDCs in the CON and UA groups and in the NST and ST groups, respectively [Figure 2(2)]. The expression levels and MFI values of moDC OVA in the ST group were lower than those in the CON and UA groups (*p* < 0.05). However, there was no statistical difference in the MFI values of antigen presentation and phagocytosis function of the moDCs of the NST, CON and UA groups [Figure 2(3)]. ELISA indicated that the expression levels of IL‐6 and IL‐12p70 in the ST and NST groups were higher than that in the CON and UA groups (*p* < 0.05) (see Supplementary data, Figure S1A,C). Regarding the expression levels of IL‐10, there was no statistically significant difference among all groups (see Supplementary data, Figure S1B).

### 3.4 Differential expression of lncRNAs

Table 2 shows the expression profiles of differentially expressed lncRNAs among the different studied groups, and inter‐group comparisons. When compared with the CON group, the highest number of lncRNAs upregulated was found in the ST group, followed by NST and UA groups, respectively. On the other hand, when compared with the UA group, the highest number of expressed lncRNAs was found in the ST group, followed by the NST group. Whereas, when NST was compared with ST, no lncRNA was found to be upregulated (Table 2). Tables 3 and 4 list the top 10 lncRNAs that were differentially expressed in comparison of patients with UA and patients with STEMI and comparison of healthy patients and patients with STEMI (Tables 3 and 4). The differentially expressed lncRNAs were showed using volcano maps (see Supplementary data, Figure S2), and their distribution is illustrated using heatmaps (see Supplementary data, Figure S3).

**TABLE 2:** Differences in the number of lncRNAs expressed among the groups.

Compare‐group	Sig	Up	Down
CON vs UA	3	1	2
CON vs NST	49	13	36
CON vs ST	35	18	17
UA vs NST	115	44	71
UA vs ST	113	70	43
NST vs ST	4	0	4

Abbreviations: CON, control, healthy volunteers; Down, significantly downregulated gene; NST, non‐ST‐segment elevation myocardial infarction; Sig, number of significantly differentially expressed genes; ST, ST‐segment elevation myocardial infarction; Up, significantly upregulated gene.

**TABLE 3:** Top 10 differentially expressed lncRNAs based on the fold change (FC) values in patients with ST versus patients with UA.

Upregulated lncRNAs		Down‐regulated lncRNAs	
lncRNA ID	Fold change	lncRNA ID	Fold change
NR_046502.1	6.847151558	NR_001564.2	−8.666432781
NR_125734.1	6.794857735	NR_003255.2	−8.599792289
NR_125733.1	6.620243097	ENST00000381108.3	−6.432797217
NR_125736.1	6.38162831	ENST00000413897.2	−5.959498631
NR_125735.1	6.36601394	ENST00000447389.1	−5.866711676
NR_125737.1	5.92137718	NR_001558.3	−4.907223863
ENST00000521498.1	5.705934793	ENST00000454861.2	−4.777113613
NR_001543.3	5.432879437	NR_037843.3	−4.601534508
ENST00000566193.1	5.410383608	NR_121625.1	−3.936075356
NR_144458.1	5.165316333	ENST00000453666.1	−3.917072598

Abbreviations: lncRNAs, long non‐coding RNAs; ST, ST‐segment elevation myocardial infarction; UA, unstable angina.

**TABLE 4:** Top 10 differentially expressed lncRNAs based on the fold change (FC) values in patients with ST versus CON.

Upregulated lncRNAs		Down‐regulated lncRNAs	
lncRNA ID	Fold change	lncRNA ID	Fold change
NR_027922.3	4.026332123	NR_001564.2	−8.11180645
NR_027921.3	3.891106436	NR_003255.2	−8.045015752
ENST00000514425.1	2.314561714	NR_001558.3	−6.842515511
ENST00000585945.1	1.903064576	ENST00000433510.2	−5.982336514
ENST00000586030.1	1.851923392	NR_037843.3	−5.18496365
ENST00000590780.1	1.822037256	NR_027701.1	−3.011757335
ENST00000609178.1	1.723826313	ENST00000608596.1	−2.327906013
ENST00000434683.1	1.700832016	NR_038885.1	−2.271360898
ENST00000609272.1	1.608128612	ENST00000491934.2	−2.056528047
ENST00000592117.1	1.596146278	ENST00000602277.1	−1.716557573

Abbreviations: CON, healthy volunteers; lncRNAs, long non‐coding RNAs; ST, ST‐elevation myocardial infarction.

Based on conservative scores and differential expression multiples, five lncRNAs, ENST00000446952.1, ENST00000457097.1, ENST00000590780.1, NR_026793.1 and NR_027922.3, were selected for quantitative reverse transcription polymerase chain reaction (qRT‐PCR) analysis. The results showed that the NR_026793.1, NR_027922.3, ENST00000446952.1 and ENST00000590780.1, were upregulated, while the expression level of ENST00000457097.1, was not significantly different between the AMI group and healthy groups (see Supplementary data, Figure S4).

The results of GO analysis demonstrated that the differentially expressed RNAs are mainly involved in cellular components; molecular functions including protein binding and enzyme binding; and biological processes including single‐organism cellular processes, cellular processes and single‐organism process (see Supplementary data, Figure S5). According to the KEGG analysis, the main signalling pathways involved were associated with metabolism and steroid hormone biosynthesis (see Supplementary data, Figure S6).

The results of Pearson's correlation coefficient showed that ENST00000590780.1 was positively correlated with CD80 (*R* = 0.61, *p* < 0.05) (see Supplementary data, Figure S7A); ENST00000446952.1 and NR_027922.3 were positively correlated with CD86 (*R* = 0.61 and *R* = 0.56; *p* < 0.05, respectively) (see Supplementary data, Figure S7B,C); NR_027922.3 and ENST00000446952.1 were positively correlated with WBC (*R* = 0.47 and *R* = 0.69; *p* < 0.05, respectively) (see Supplementary data, Figure S7D,E); NR_027922.3 and ENST00000446952.1 were positively correlated with CK‐MB (*R* = 0.60 and *R* = 0.63; *p* < 0.05, respectively) (see Supplementary data, Figure S7F,G); and NR_027922.3 and ENST00000446952.1 were positively correlated with TNT‐hs (*R* = 0.66 and *R* = 0.58; *p* < 0.05, respectively) (see Supplementary data, Figure S7H,I).

### 3.5 Role and mechanism of lncRNA C‐C motif chemokine ligand (CCL)15‐CCL14

#### 3.5.1 Lentiviral transfection

Immunofluorescence results showed that a multiplicity of infection (MOI) of 60 and an infection time of 48 h were the best conditions for lentiviral moDC transfection (see Supplementary data, Figure S8). The amplification and dissolution curves of CCL15‐CCL14 (see Supplementary data, Figure S9A–C) and RT‐PCR showed that, compared with the CON immature DC (imDC) and lentiviral empty vector groups (imDC‐vector), overexpression of the lentiviral vector upregulates the expression of CCL15‐CCL14 (*p* < 0.05) (see Supplementary data, Figure S9D).

#### 3.5.2 Effect of CCL15‐CCL14 transfection

RT‐PCR demonstrated that there was a significant increase in expression level of CCL15‐CCL14 in mature dendritic cells (mDCs) (*p* < 0.05) (see Supplementary data, Figure S10), whereas fluorescence microscopy and RT‐PCR demonstrated that the expression level of CCL15‐CCL14 in moDCs decreased after transfection with CCL15‐CCL14 (see Supplementary data, Figure S11).

#### 3.5.3 Effect of CCL15‐CCL14 on DCs

**FIGURE 3:**
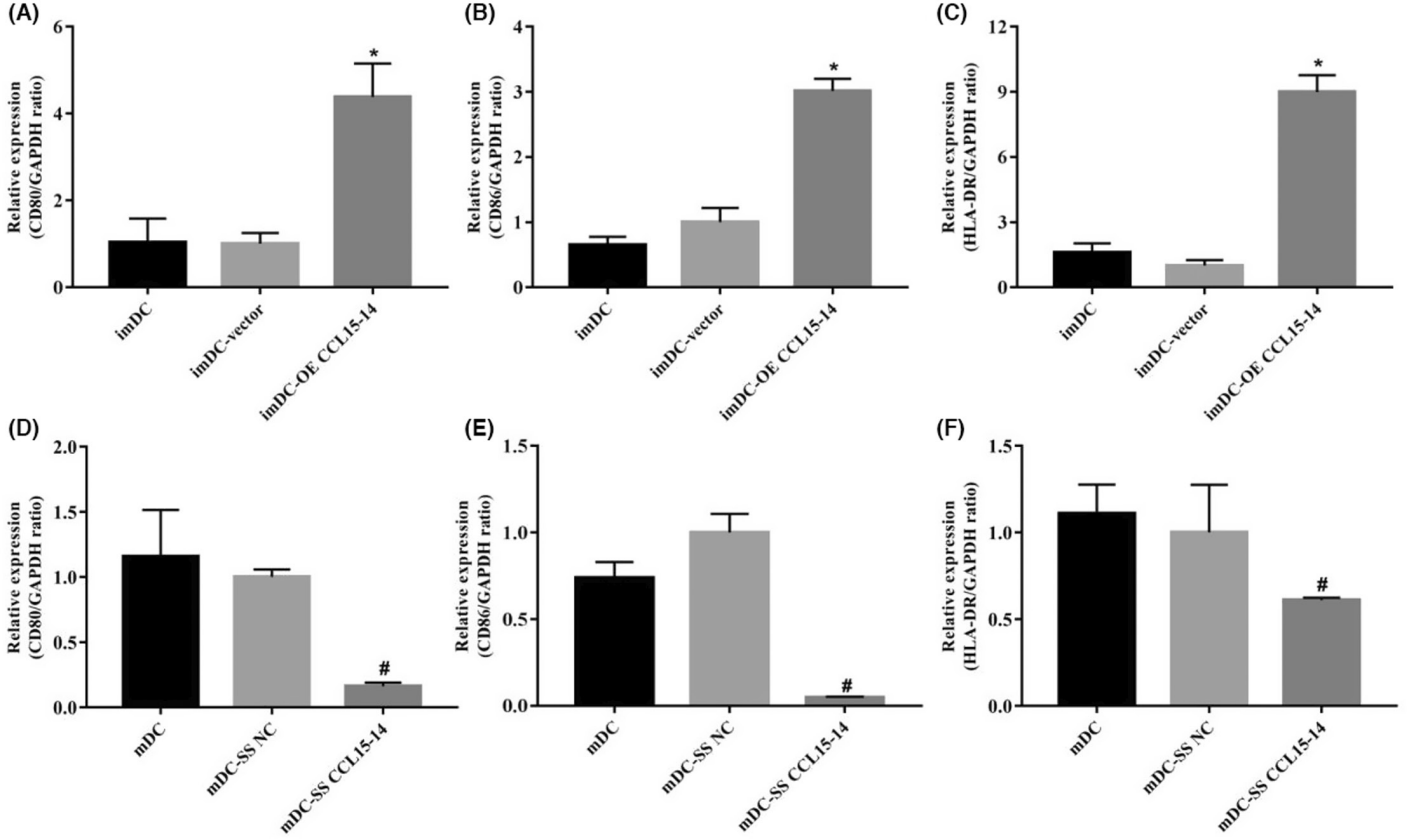
The reverse transcription‐PCR outcomes: Expression levels of markers on dendritic cell surfaces. (A–C) The expression levels of CD80, CD86, and HLA‐DR after CCL15‐CCL14 overexpression, respectively. (D–F) The expression levels of CD80, CD86, and HLA‐DR after CCL15‐CCL14 silencing, respectively. *n* = 3 *p < 0.05, compared with the imDC group; #*p* < 0.05, compared with the mDC group. imDC, immature dendritic cells; mDC‐vector, mature dendritic cells.

**FIGURE 4:**
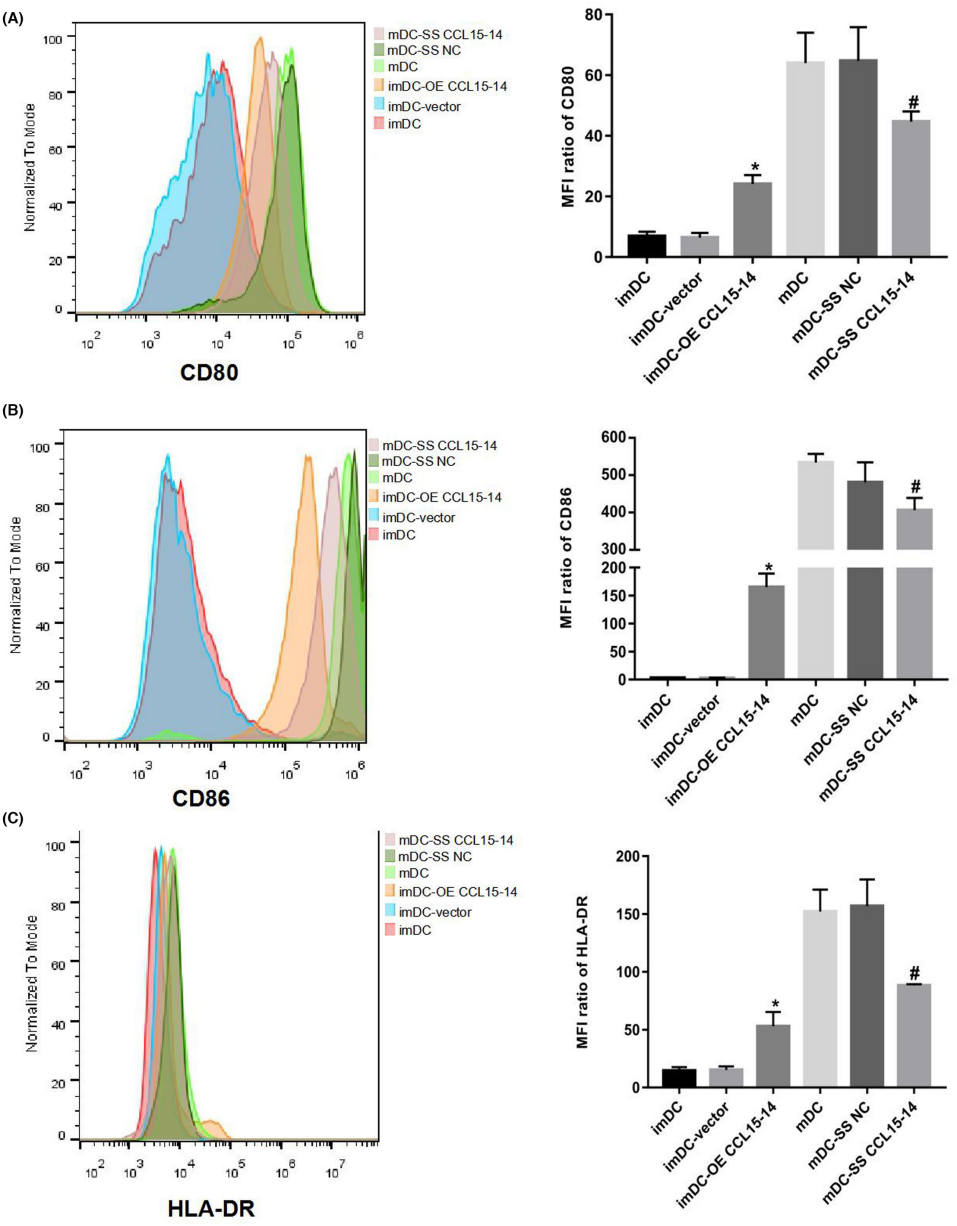
The flow cytometry results: Expression of markers on dendritic cell surfaces. (A) The expression levels of CD80 after CCL15‐CCL14 overexpression or silencing; (B): The expression levels of CD86 after CCL15‐CCL14 overexpression or silencing; (C) The expression levels of HLA‐DR after CCL15‐CCL14 overexpression or silencing. *n* = 3 *p < 0.05, compared with the imDC group; #*p* < 0.05, compared with the mDC group. MFI, mean fluorescence intensity; imDC, immature dendritic cells; mDC‐vector, mature dendritic cells.

**FIGURE 5:**
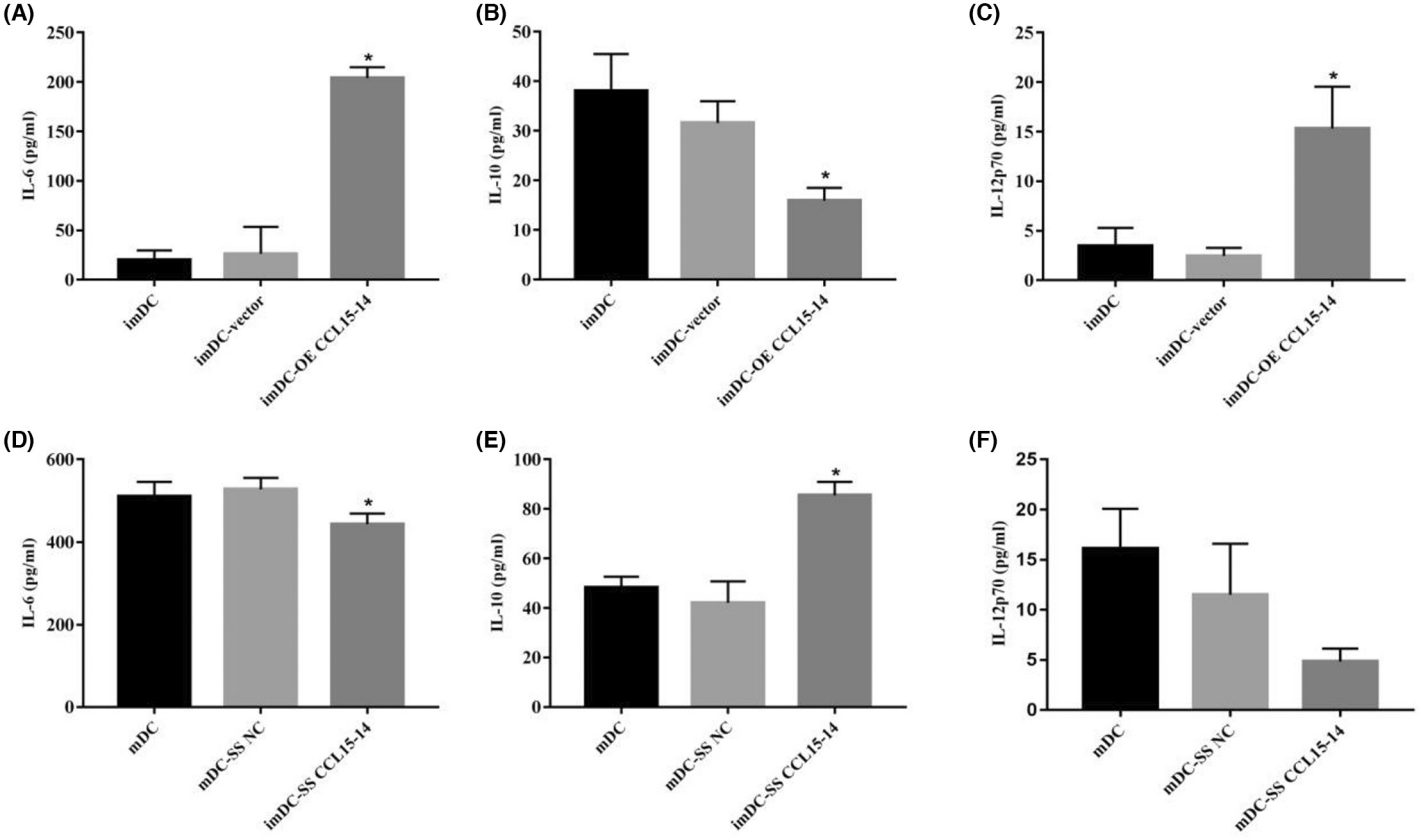
The secretory function of the dendritic cells by enzyme‐linked immunosorbent assay. (A) The expression levels of imDC IL‐6; (B) The expression levels of imDC IL‐10; (C) The expression levels of imDC IL‐12p70; (D) The expression levels of mDC IL‐6; (E) The expression levels of mDC IL‐10; (F) The expression levels of mDC IL‐12p70. *n* = 3 *p < 0.05, compared with imDC/mDC group. imDC, immature dendritic cells; mDC‐vector, mature dendritic cells.

RT‐PCR and FCM showed that the expression levels of CD80, CD86 and HLA‐DR increased after CCL15‐CCL14 overexpression; conversely, the expression levels of CD80, CD86 and HLA‐DR decreased after CCL15‐CCL14 silencing (Figures 3 and 4). ELISA showed that the expression levels of IL‐6 and IL‐12p70 increased and the expression levels of IL‐10 decreased following overexpression of CCL15‐CCL14. In contrast, the expression levels of IL‐6 and IL‐12p70 decreased and those of IL‐10 increased after CCL15‐CCL14 silencing (Figure 5).

#### 3.5.4 Pathway proteins of lncRNAs

**FIGURE 6:**
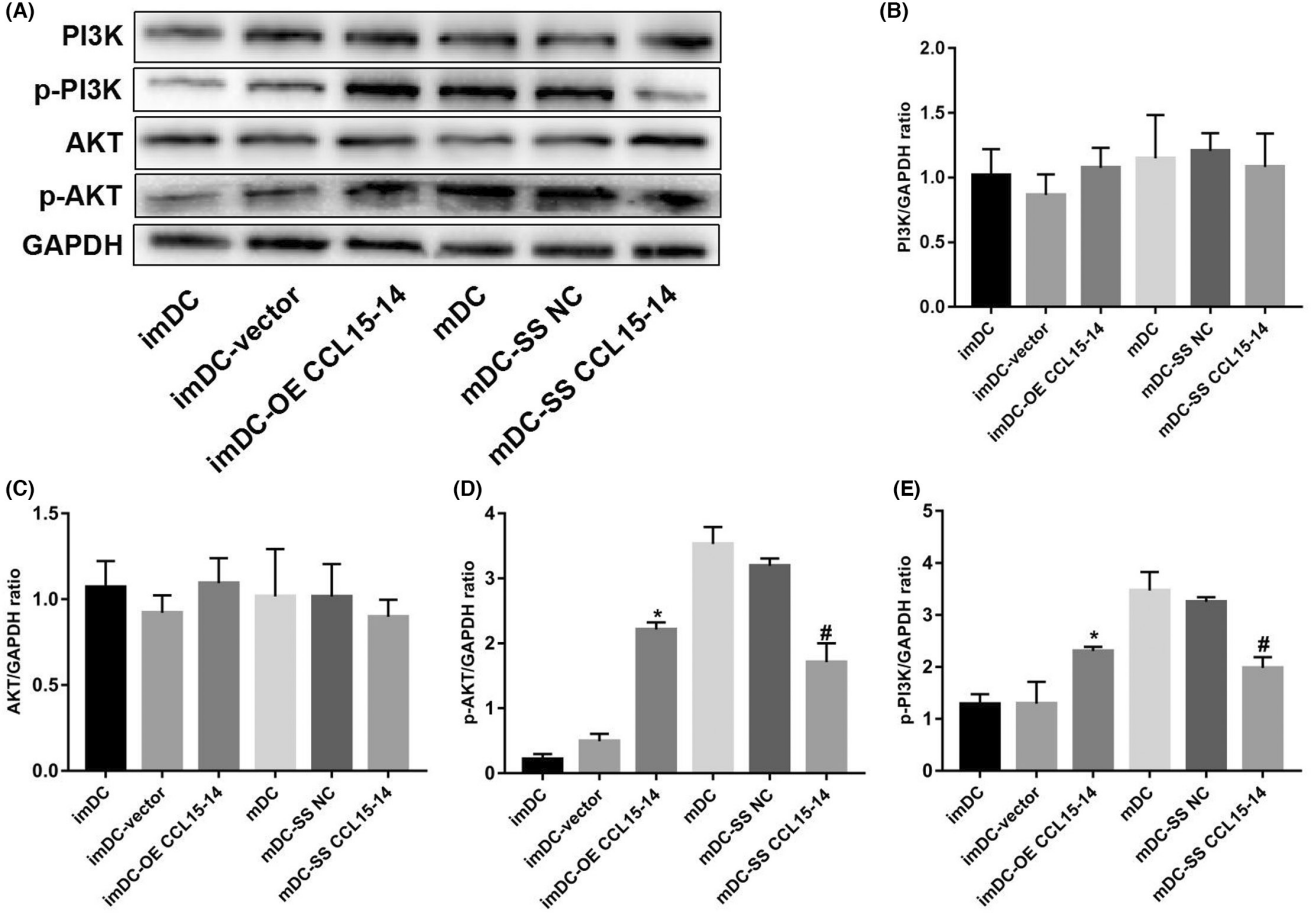
Western blot analysis of the role of CCL15‐CCL14 in signalling pathways. (A) The representative images by Western blot assay; (B) The results of PI3K; (C) The results of AKT; (D) The results of p‐AKT; (E) The results of p‐PI3K. *n* = 3 *p < 0.05, compared with the imDC group; #*p* < 0.05, compared with the mDC group. MFI, mean fluorescence intensity; imDC, immature dendritic cells; mDC‐vector, mature dendritic cells.

WB indicated that the overexpression and silencing of CCL15‐CCL14 upregulated and downregulated the expression levels of p‐PI3K and p‐AKT, respectively (Figure 6).

#### 3.5.5 lncRNA subcellular localization

Fluorescence in situ hybridization showed that CCL15‐CCL14 was mostly expressed in the nuclei of moDCs (see Supplementary data, Figure S12).

#### 3.5.6 Regulatory effect of lncRNA CCL15‐CCL14 on CCL14

**FIGURE 7:**
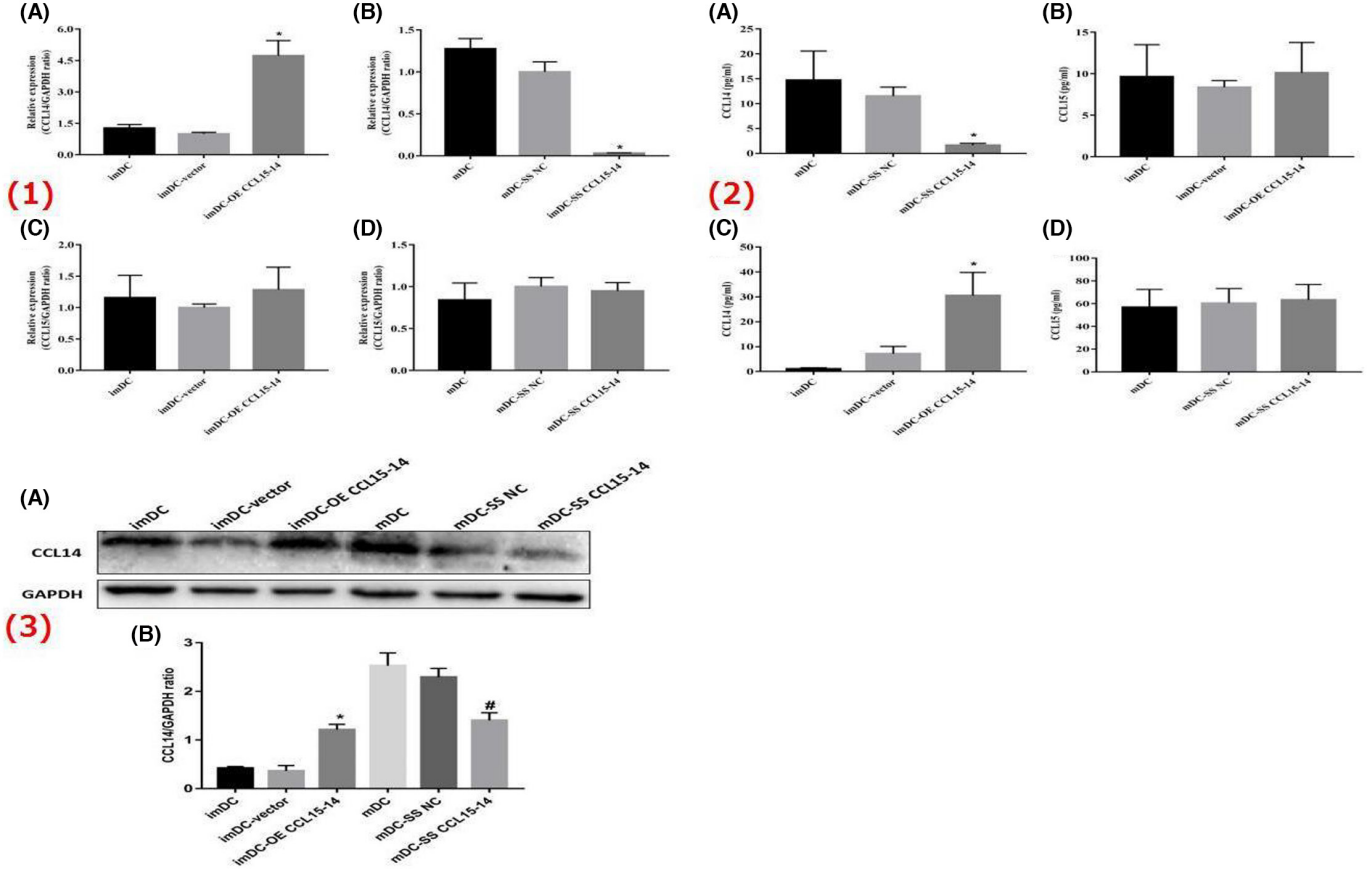
The expression levels of moDCs' derived CCL14 and CCL15 in each group. (1)The results of reverse transcription‐PCR. (A) The results of imDCs' derived CCL14; (B) The results of mDCs' derived CCL14; (C) The results of imDCs' derived CCL15; (D) The results of mDCs' derived CCL15; (2)The results of ELISA. (A) The expression levels of mDCs' derived CCL14; (B) The expression levels of imDCs' derived CCL15; (C) The expression levels of imDCs' derived CCL14; (B) The expression levels of mDCs' derived CCL15; (3) The results of Western blot assay. (A) The representative images by Western blot assay; (B) The results of CCL15‐CCL14 after overexpression or silencing. *n* = 3 *p < 0.05, compared with the imDC group; #*p* < 0.05, compared with the mDC group; ELISA, enzyme‐linked immunosorbent assay; C–C motif chemokine ligand (CCL); GAPDH, glyceraldehyde‐3‐phosphate dehydrogenase; imDC, immature dendritic cells; mDC‐vector, mature dendritic cells.

The RT‐PCR, ELISA and WB demonstrated that the overexpression of CCL15‐CCL14 upregulated the expression of CCL14. In contrast, silencing CCL15‐CCL14 downregulated the expression of CCL14. However, the expression level of CCL15 did not change with the expression/silencing of lncRNA CCL15‐CCL14 (Figure 7).

## 4 DISCUSSION

This study aimed to clarify the relationship between the pathogenesis of ACS and gene expression, especially lncRNAs. To our knowledge, this study is the first to explore moDC morphology and function, differences in moDC‐derived lncRNA expression and the influence of differentially expressed lncRNAs in moDCs in patients with ACS.

In this study, we found that in patients with different types of ACS: (1) the moDC morphology and function were different—moDCs in patients with acute myocardial infarction (AMI) had a stronger antigen presenting capability and pro‐inflammatory secretion function and a weaker phagocytic capability; and (2) the lncRNAs of moDC‐derived lncRNAs differentially expressed between patients with myocardial infarction (NSTEMI and STEMI) and patients with non‐myocardial infarction (UA and CON). However, fewer lncRNAs were differentially expressed between the CON and UA groups and between the NSTEMI and STEMI groups; and (3) differentially expressed lncRNAs (CCL15‐CCL14) have regulatory effects on moDCs.

Rather than focusing on the role of any specific lncRNA in ACS, the primary focus was whether there were variations in lncRNA expression in patients with ACS. Yang et al.^13^ reported that 679 and 570 lncRNAs were differentially expressed in the ischemic group and the non‐ischemic group, respectively, when they sequenced the genes of ventricular tissues. Furthermore, they believed that lncRNAs, rather than mRNAs or miRNAs, were involved in heart failure and ventricular remodelling. Zhong et al.^14^ and Zhao et al.^15^ reported that 58 lncRNA were differentially expressed between patients with STEMI and NSTEMI and 106 lncRNA between patients with AMI and non‐coronary patients when circulating monocytes were sequenced, respectively. Whereas, Sheng et al.^16^ showed that 552 lncRNAs were differentially expressed between patients with AMI and healthy participants when circulating endothelial cells were sequenced. The results of the present study showed that patients with different types of ACS expressed different lncRNAs. The highest number of differentially expressed lncRNA was observed when comparing the UA group with the ST group (113), followed by UA versus NST (115), CON versus NST (49) and CON versus ST (35). However, the number of lncRNAs when comparing the CON group with the UA group (3) and the ST group with the NST group (4) was low.

Several studies have found that different types of ACS are correlated with the differential expression of lncRNAs. Additionally, the number of differentially expressed lncRNAs in patients with ACS vary greatly among several studies, which may be due to the different sources of lncRNAs (monocytes, endothelial cells and cardiomyocytes).

Another issue that has to be addressed is: which tissue‐ or cell‐derived lncRNAs are crucial for the occurrence and progression of ACS?

In the present study, moDCs were selected as the source of lncRNAs for the following three reasons: (1) DCs are closely related to the development of atherosclerosis and ACS^17^; (2) DCs, as important components of the blood, have an important role in systemic regulation^18^, ^19^; (3)different DC subgroups have significant differences in their functions. Currently, DCs are divided into four subgroups^20^: conventional dendritic cells (cDCs), mainly involved in antigen presentation and activation of T lymphocytes and TH cells to induce cellular and humoral immune effects; plasmacytoid dendritic cells (pDCs), known for producing high doses of type I interferons and thus exert antiviral immunity; Langerhans cells (LCs), mainly involved in immunological defence role in the epidermis; and moDCs, involved in the occurrence and progression of inflammatory diseases.^20^ The fact that ACS is a type of inflammatory disease, made us select moDCs as the target molecule for exploring the relationship between moDC‐derived lncRNAs and ACS.

Furthermore, the results of this study showed that moDC morphology and function were different in patients with different types of ACS; moDCs in patients with AMI had a stronger antigen presenting capability and pro‐inflammatory secretion function and a weaker phagocytosis ability, which was consistent with the results of gene sequencing.

NR_027922.3, also known as CCL15‐CCL14, is a read‐through transcript that spans both *CCL14* and *CCL15* genes. Based on the fold change in gene sequencing (fold change: 4.026332123) and RT‐PCR results (among the five candidate lncRNAs, NR_027922.3 had the highest expression level), CCL15‐CCL14 was selected for studying the influence on moDCs and related mechanisms. The results showed that lncRNA CCL15‐14 regulates the expression levels of DC markers (CD80, CD86 and HLA‐DR), inflammatory factors (IL‐6 and IL‐12p70) and target genes (*CCL14*). Additionally, it affects the phosphorylation of the PI3K/AKT signalling pathway. CCL15 and CCL14 are chemokines involved in immune cell recruitment and inflammation. They play a role in various inflammatory conditions and have been implicated in the pathogenesis of diseases such as rheumatoid arthritis, asthma and atherosclerosis.^21^ Studies have shown that CCL14 is upregulated in DCs, promotes the activation of macrophages and phosphorylation of the PI3K/AKT signalling pathway and positively regulates the immune response.^22^ Combined with the results of the present study, we believe that CCL15‐CCL14 activates DCs to participate in the development of ACS by regulating CCL14 expression and mediating the phosphorylation of the PI3K/AKT pathway.

Notably, the number of moDC‐derived lncRNAs differentially expressed between patients with UA and patients with MI was the highest, even exceeding the number of lncRNAs between controls and patients with MI, in our study. This may be because patients with UA or MI are in pathological states with many influencing factors; however, controls have relatively normal physiological states and fewer influencing factors. Therefore, it can be inferred that lncRNAs that are differentially expressed between healthy individuals and patients with MI are more specific and valuable.

In conclusion, the pathogenesis of ACS, as a global health concern, has not been fully elucidated. As for the pathogenesis of ACS, more studies have focused on local mechanisms related to plaque characteristics and environmental factors, while few studies have focused on the systemic mechanisms of gene expression and immune. This study showed that the factors of gene expression and immune were related to the pathogenesis of ACS. Therefore, regarding the pathogenesis of ACS, this study provides reference and new perspective from the level of immunity and gene expression.

## 5 LIMITATIONS

This study had a few limitations. First, this study on the relationship between the pathogenesis of ACS and gene expression, especially lncRNAs, are primarily descriptive. However, based on the important role of gene expression and dendritic cells in the pathogenesis of ACS, we believe that this study is valuable and contributes to the existing body of knowledge in the area of long noncoding RNAs in immune cell function. We will further explore the role and mechanism of dendritic cell‐derived lncRNAs in ACS in detail in the future. Second, the sample size was small. Third, the relationship between CCL15‐CCL14 and endothelial cells was not elucidated.

## 6 CONCLUSION

The morphology, function and expression of lncRNA of moDCs differ among patients with ACS depending on the type of ACS. Furthermore, the differentially expressed lncRNA, CCL15‐CCL14, regulates moDCs. These findings may help in comprehending the molecular mechanism of ACS. Understanding the role of lncRNAs in ACS may provide new insights into disease mechanisms and identification of novel diagnostic markers and therapeutic targets. Further research is needed to elucidate the specific lncRNAs involved in ACS and their mechanisms of action.

## AUTHOR CONTRIBUTIONS

**Zhenglong Wang:** Conceptualization (lead); data curation (lead); formal analysis (lead); funding acquisition (lead); investigation (lead); methodology (supporting); project administration (lead); resources (lead); software (supporting); supervision (equal); validation (lead); visualization (lead); writing – original draft (lead). **Chaohao Ke:** Conceptualization (equal); data curation (equal); formal analysis (equal); funding acquisition (supporting); investigation (equal); methodology (equal); project administration (equal); resources (supporting); software (equal); supervision (supporting); validation (equal); visualization (equal); writing – original draft (supporting). **Yuheng Chen:** Conceptualization (equal); data curation (equal); formal analysis (equal); funding acquisition (equal); investigation (equal); methodology (equal); project administration (equal); resources (equal); software (equal); supervision (equal); validation (equal); visualization (equal); writing – original draft (equal). **Yongchao Zhao:** Conceptualization (supporting); data curation (supporting); formal analysis (supporting); funding acquisition (supporting); investigation (supporting); methodology (supporting); project administration (supporting); resources (supporting); software (supporting); supervision (supporting); validation (supporting); visualization (supporting); writing – original draft (supporting). **Yuanjie He:** Conceptualization (equal); data curation (equal); formal analysis (equal); funding acquisition (equal); investigation (equal); methodology (equal); project administration (equal); resources (equal); software (equal); supervision (equal); validation (equal); visualization (equal); writing – original draft (equal). **Xiao Liang:** Conceptualization (supporting); data curation (supporting); formal analysis (supporting); funding acquisition (supporting); investigation (supporting); methodology (supporting); project administration (supporting); resources (supporting); software (supporting); supervision (supporting); validation (supporting); visualization (supporting); writing – original draft (supporting). **Qingxian Tu:** Conceptualization (supporting); data curation (supporting); formal analysis (supporting); funding acquisition (supporting); investigation (supporting); methodology (supporting); project administration (supporting); resources (supporting); software (supporting); supervision (supporting); validation (supporting); visualization (supporting); writing – original draft (supporting). **Min Xu:** Conceptualization (supporting); data curation (supporting); formal analysis (supporting); funding acquisition (supporting); investigation (supporting); methodology (supporting); project administration (equal); resources (supporting); software (supporting); supervision (supporting); validation (supporting); visualization (supporting); writing – original draft (supporting). **Fujia Guo:** Conceptualization (equal); data curation (equal); formal analysis (equal); funding acquisition (equal); investigation (equal); methodology (equal); project administration (equal); resources (equal); software (equal); supervision (equal); validation (equal); visualization (equal); writing – original draft (equal). **Junbo Ge:** Conceptualization (equal); data curation (supporting); formal analysis (supporting); funding acquisition (supporting); investigation (supporting); methodology (supporting); project administration (supporting); resources (supporting); software (supporting); supervision (equal); validation (supporting); visualization (supporting); writing – original draft (supporting). **Bei Shi:** Conceptualization (equal); data curation (equal); formal analysis (equal); funding acquisition (equal); investigation (equal); methodology (equal); project administration (equal); resources (equal); software (equal); supervision (equal); validation (equal); visualization (equal); writing – original draft (supporting).

## FUNDING INFORMATION

This work was funded by the Regional Fund Project of the National Natural Science Foundation of China (project name: Differential expression of DC‐derived lncRNAs in acute coronary syndrome and their regulatory mechanism; project number: 81760072).

## CONFLICT OF INTEREST STATEMENT

None.

## Supporting information


Data S1.

## Data Availability

The data presented in this article can be obtained from the corresponding author upon reasonable request.
