# Supplementary material for: Dendritic cell‐derived lncRNAs in patients with acute coronary syndrome
Source: J Cell Mol Med. 2024 Oct 21;28(20):e70057. doi: 10.1111/jcmm.70057 (PMC11493550; doi:10.1111/jcmm.70057)
Supplement: Supplementary file 1 — Data S1. [file JCMM-28-e70057-s001.doc]

**Supplementary data**

| **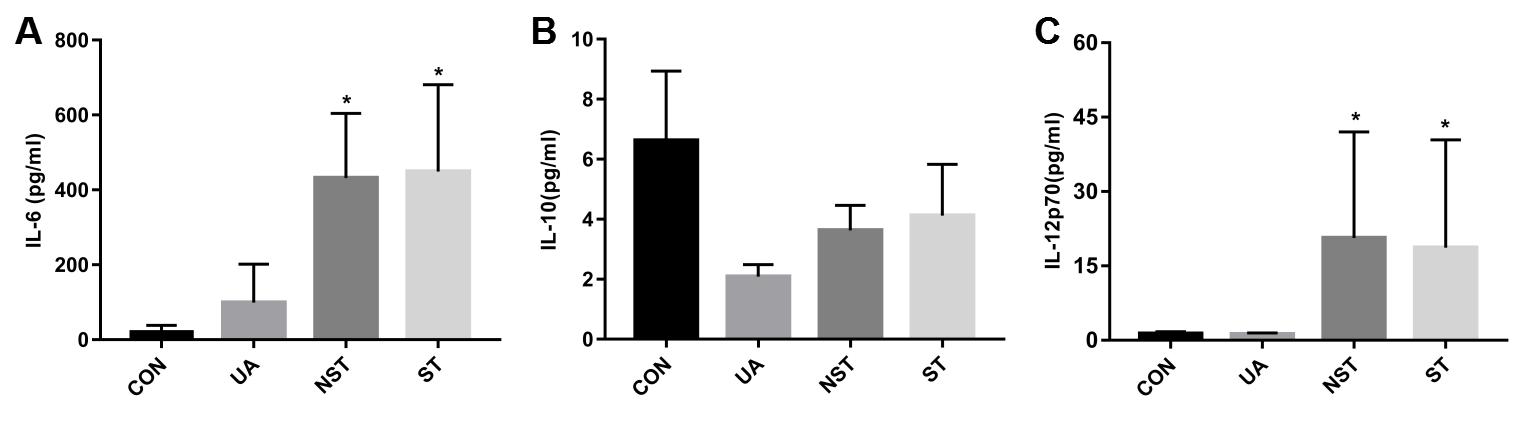** |
| --- |

**Figure S1 The ELISA results of interleukin secretion by moDCs among the groups**

Note: *p<0.05, compared with CON.

moDCs, monocyte-derived dendritic cells; UA, unstable angina; NST, non-ST-segment elevation myocardial infarction; ST, ST-segment elevation myocardial infarction; CON, normal control.

| 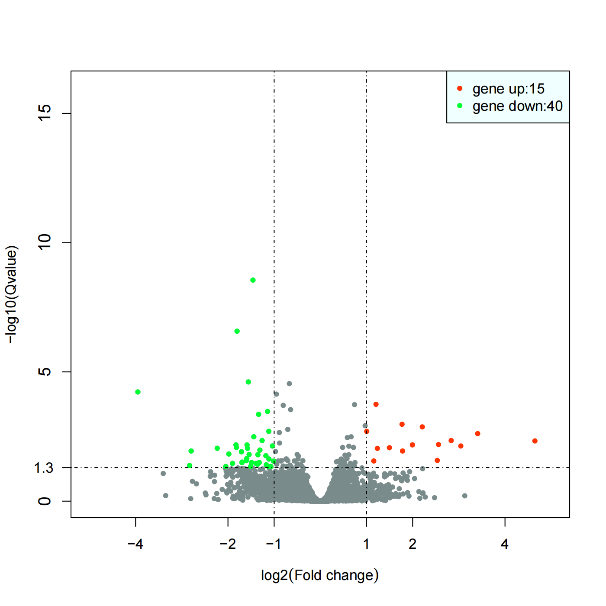  **A** | 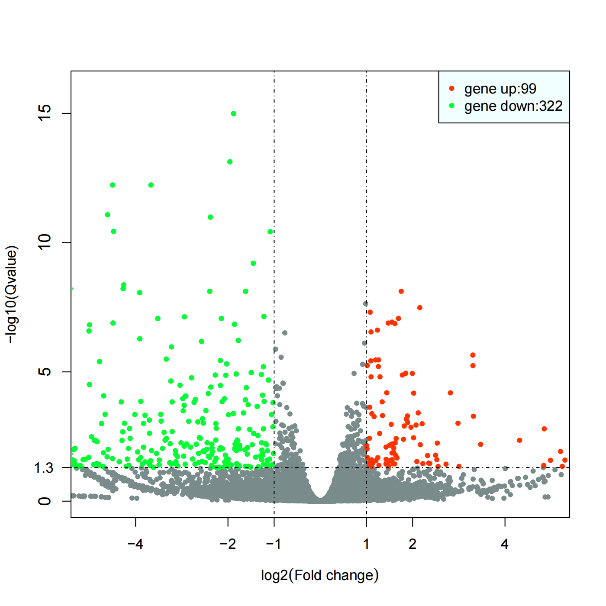  **B** |
| --- | --- |
| 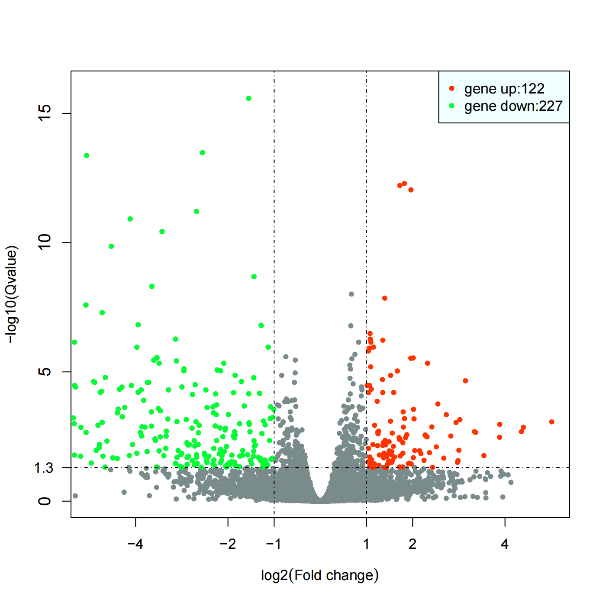  **D**  **C** | 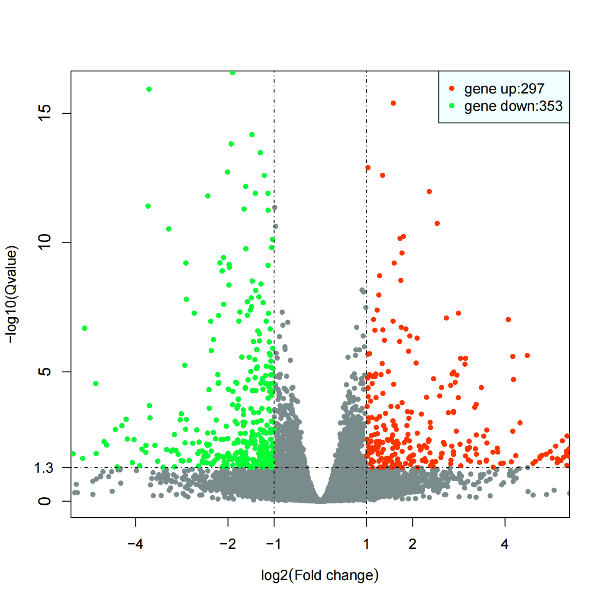 |
| 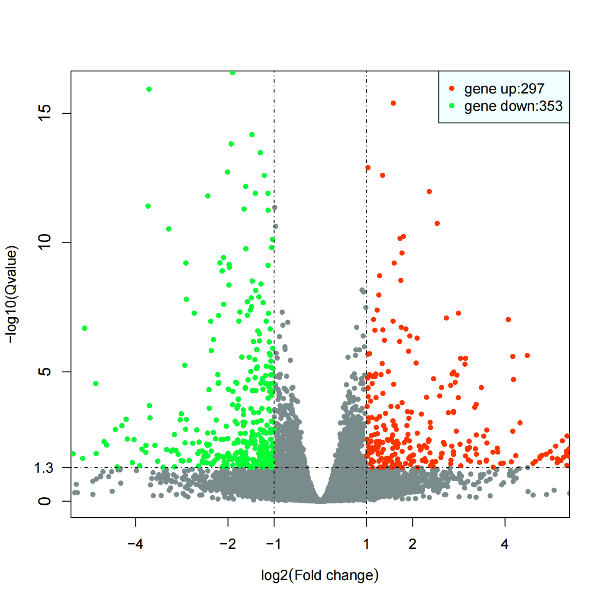  **E** | 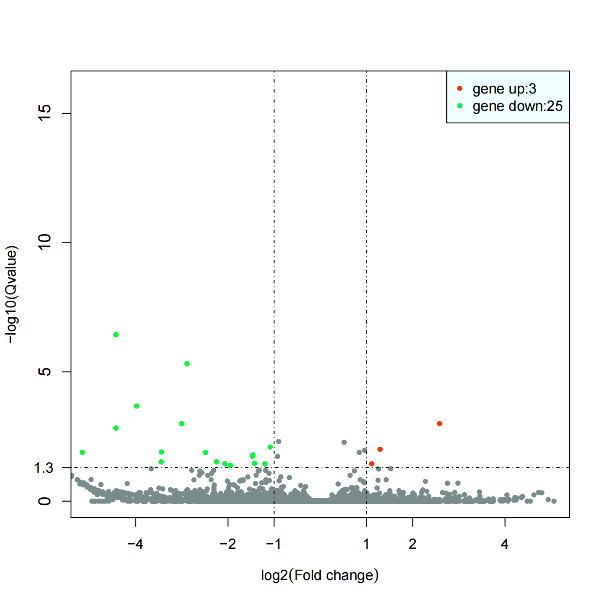  **F** |

**Figure S2. Volcano plots depicting differential expression of lncRNAs in different groups**

Red dots represent upregulated lncRNAs and green dots represent downregulated lncRNAs and mRNAs with statistical significance (fold change ≥2, *P* < 0.05), while the gray dots are not statistically significant (*P* > 0.05). **A**, CON vs. UA (n=3); **B**, CON vs. NST (n=3); C, CON vs ST (n=3); D, UA vs NST (n=3); E, UA vs ST (n=3); F, NST vs ST (n=3). LncRNAs, long non-coding ribonucleic acids; CON, controls, healthy volunteers; UA, unstable angina; NST, non-ST-segment elevation myocardial infarction

| 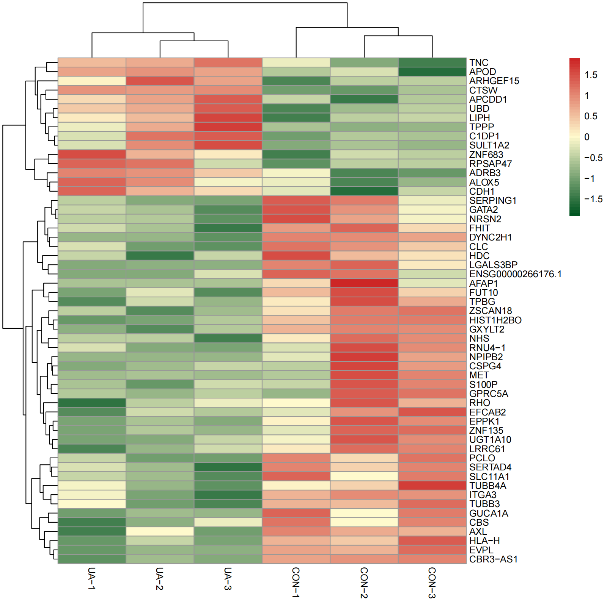  **UA(n=3)**  **CON(n=3)**  **A** | 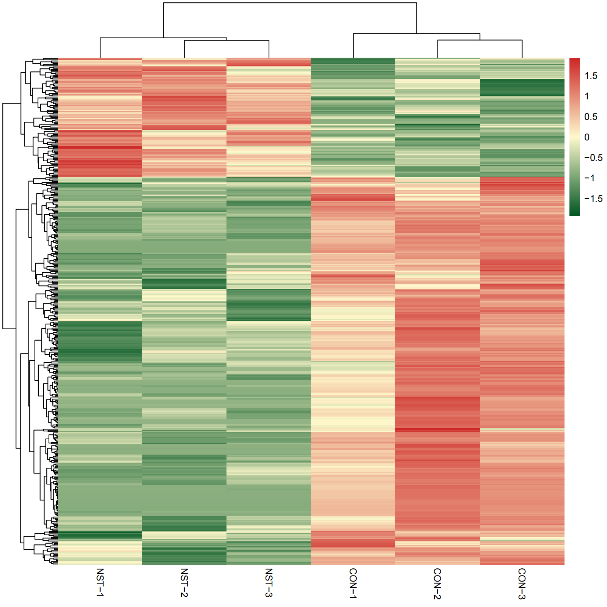  **NST(n=3)**  **CON(n=3)**  **B** |
| --- | --- |
| 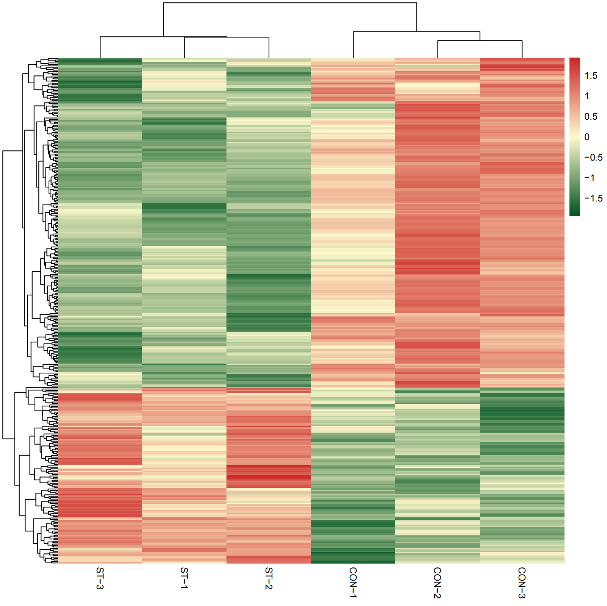  **D**  **C**  **ST(n=3)**  **CON(n=3)** | 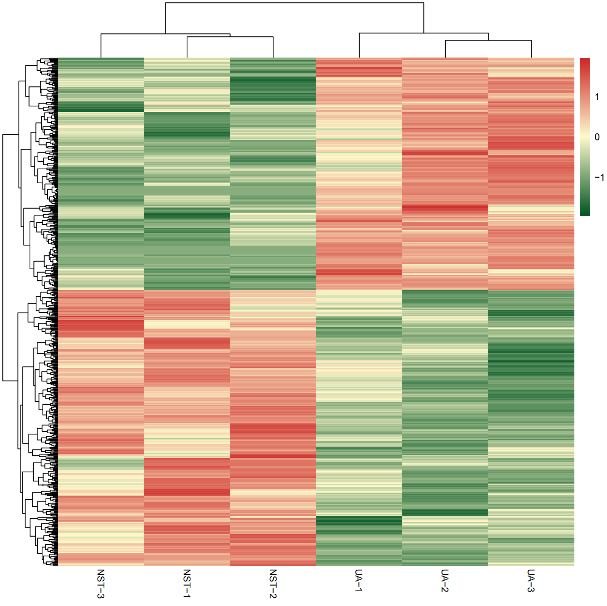  **NST(n=3)**  **UA(n=3)** |
| 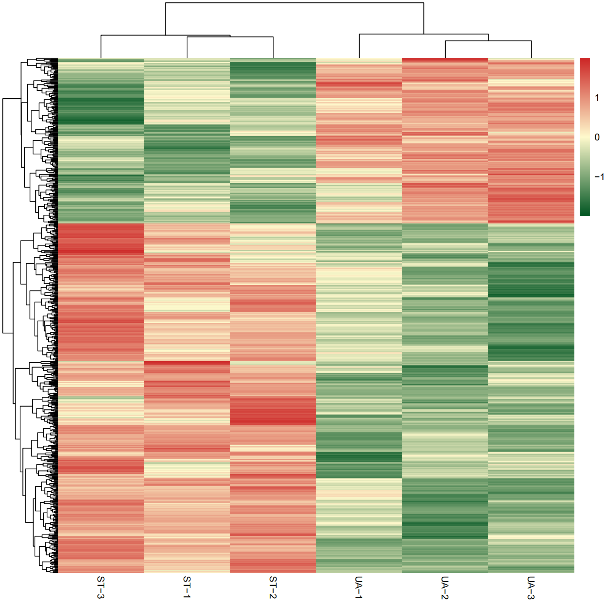  **ST(n=3)**  **UA(n=3)**  **E** | 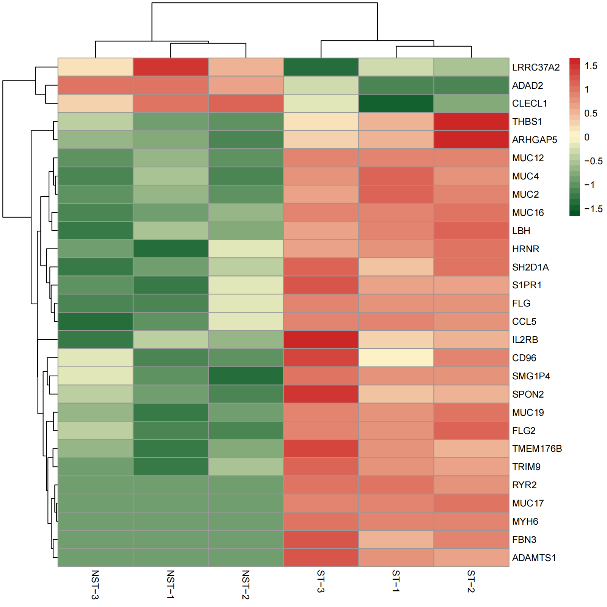  **NST(n=3)**  **ST(n=3)**  **F** |

**Figure S3. Heatmaps of differential expression of lncRNAs in different groups**

Red and green colors indicate high and low relative expression, respectively. LncRNA, long non-coding ribonucleic acid; UA, unstable angina; NST, non-ST-segment elevation myocardial infarction; ST, ST=segment elevation myocardial infarction

| **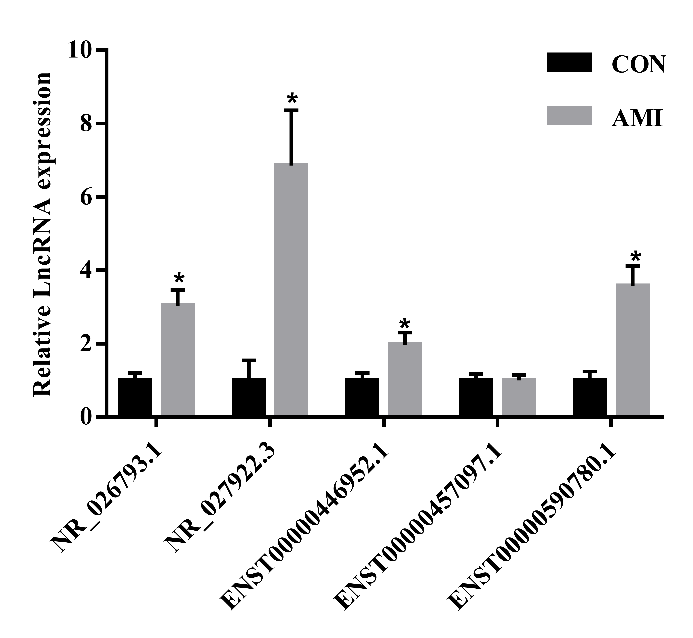** |
| --- |

**Figure S4. Sequencing results verified by RT-PCR**

AMI: n=14, CON: n=5; *p<0.05, compared with CON.

LncRNA, long non-coding ribonucleic acid; AMI, acute myocardial infarction; CON, controls, healthy volunteers

| **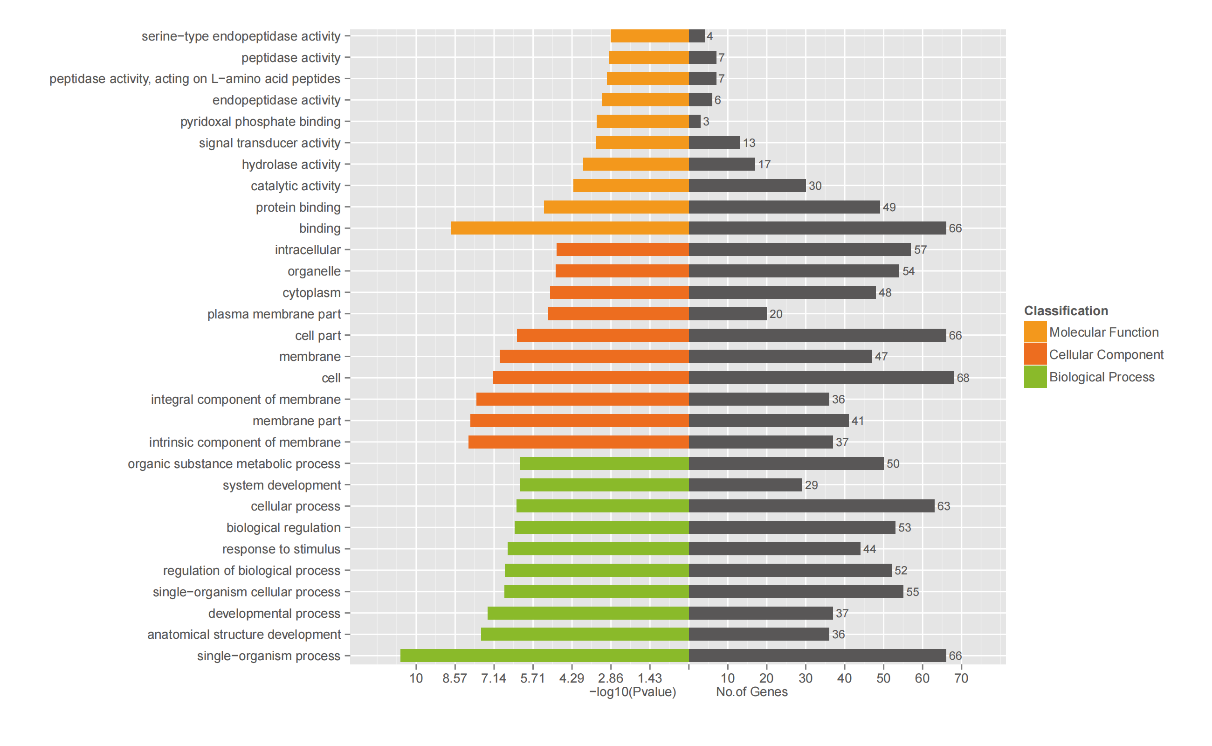**  **A** |
| --- |
| **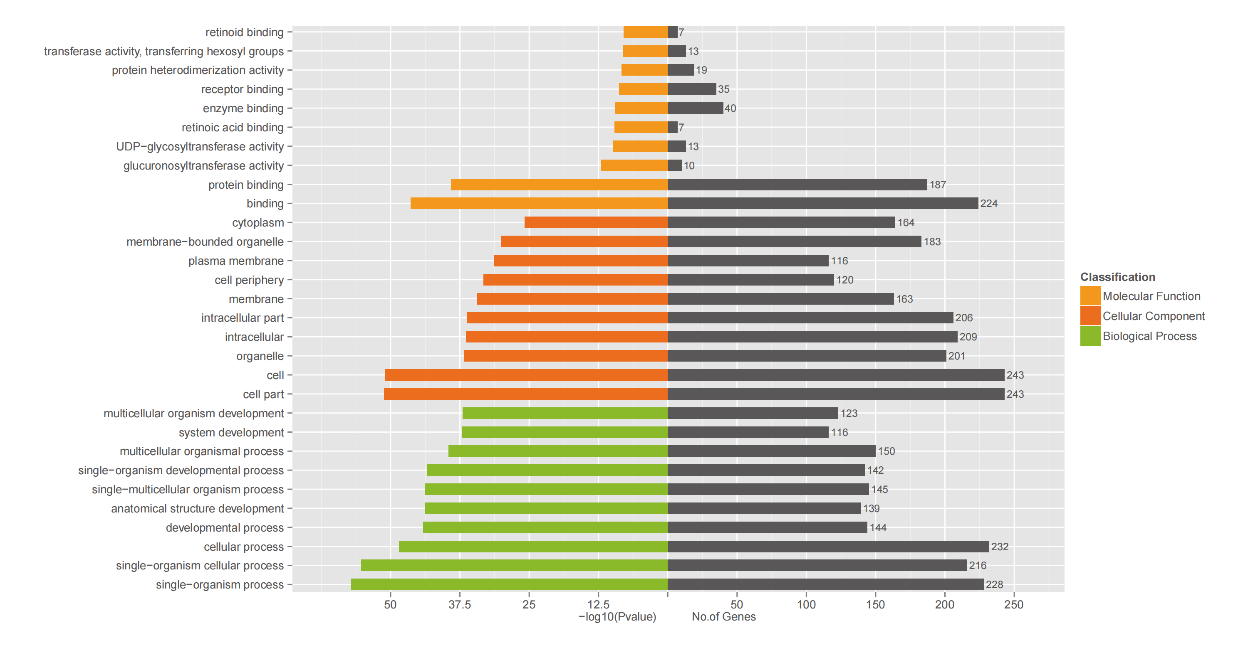**  **B** |
| 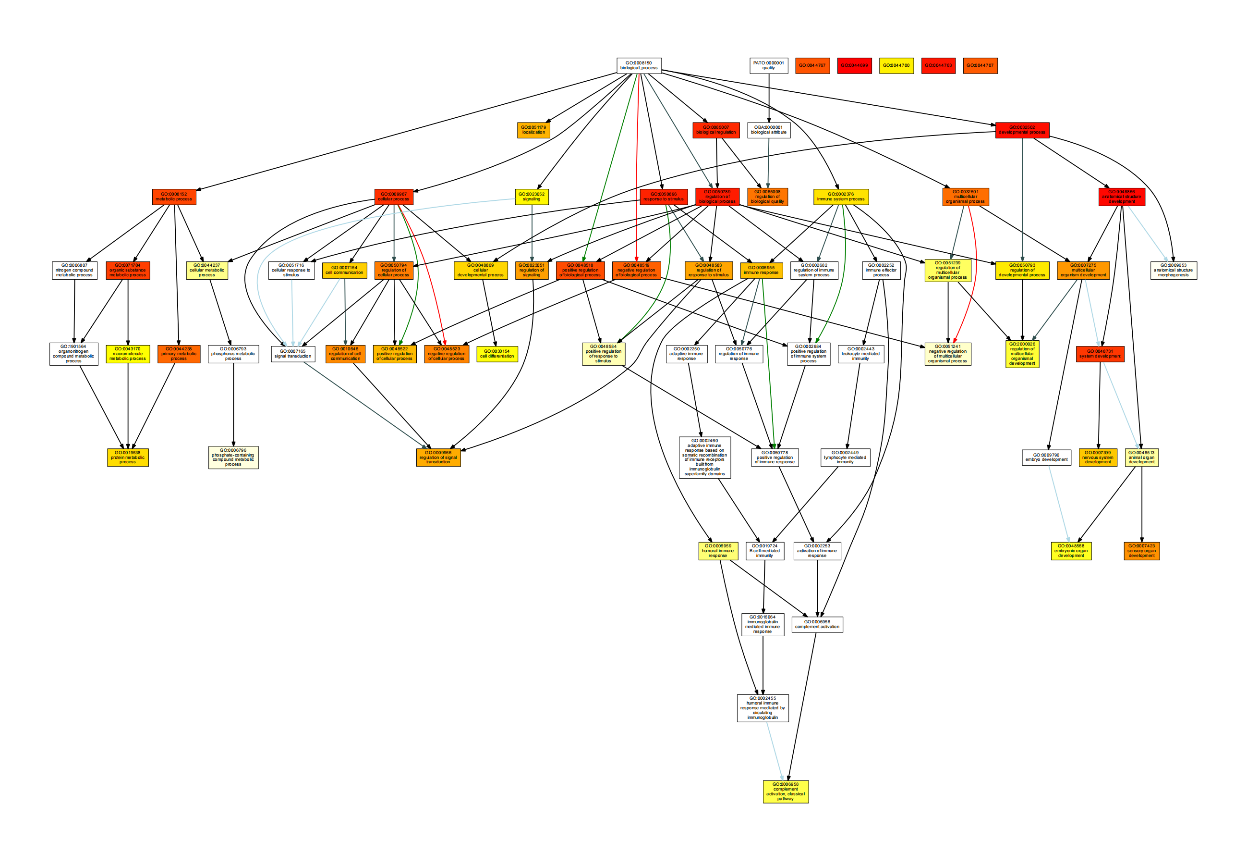  **C** |
| 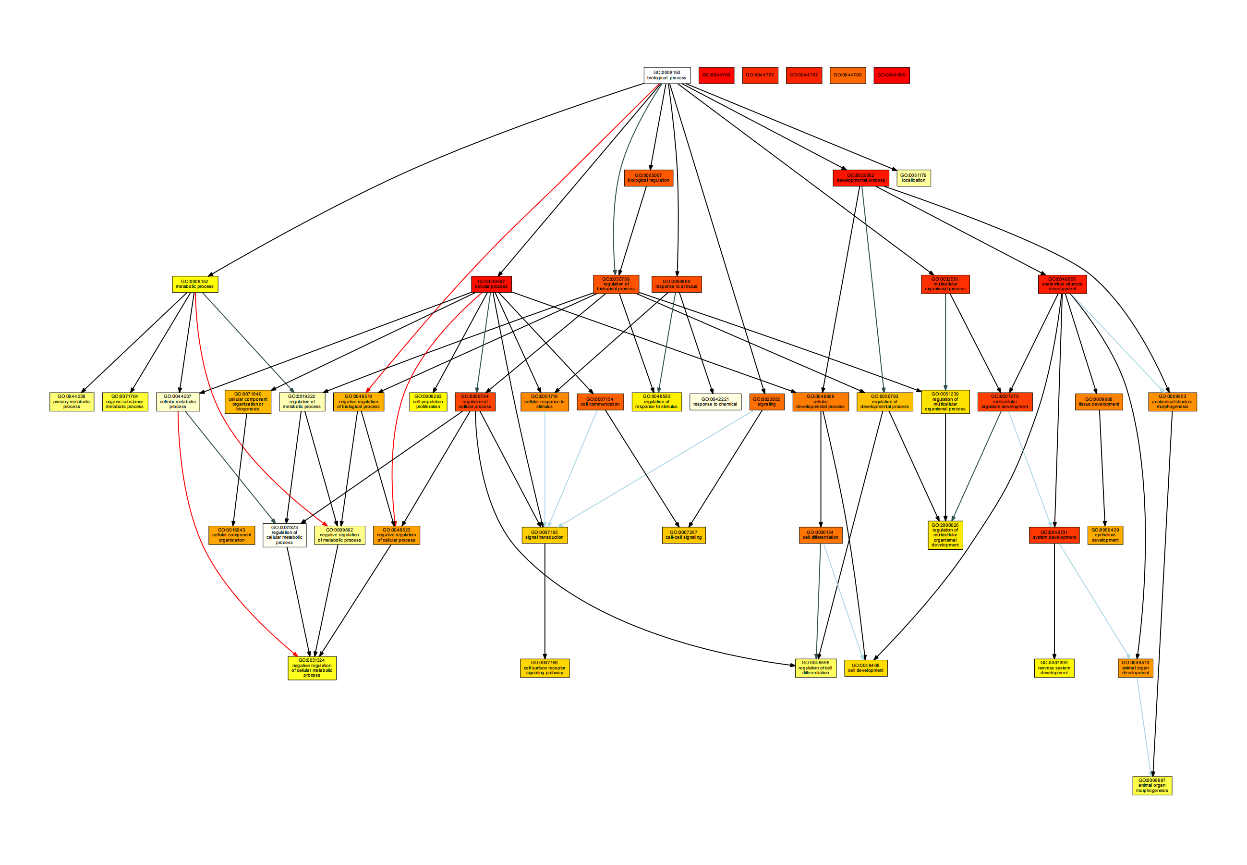  **D** |

| 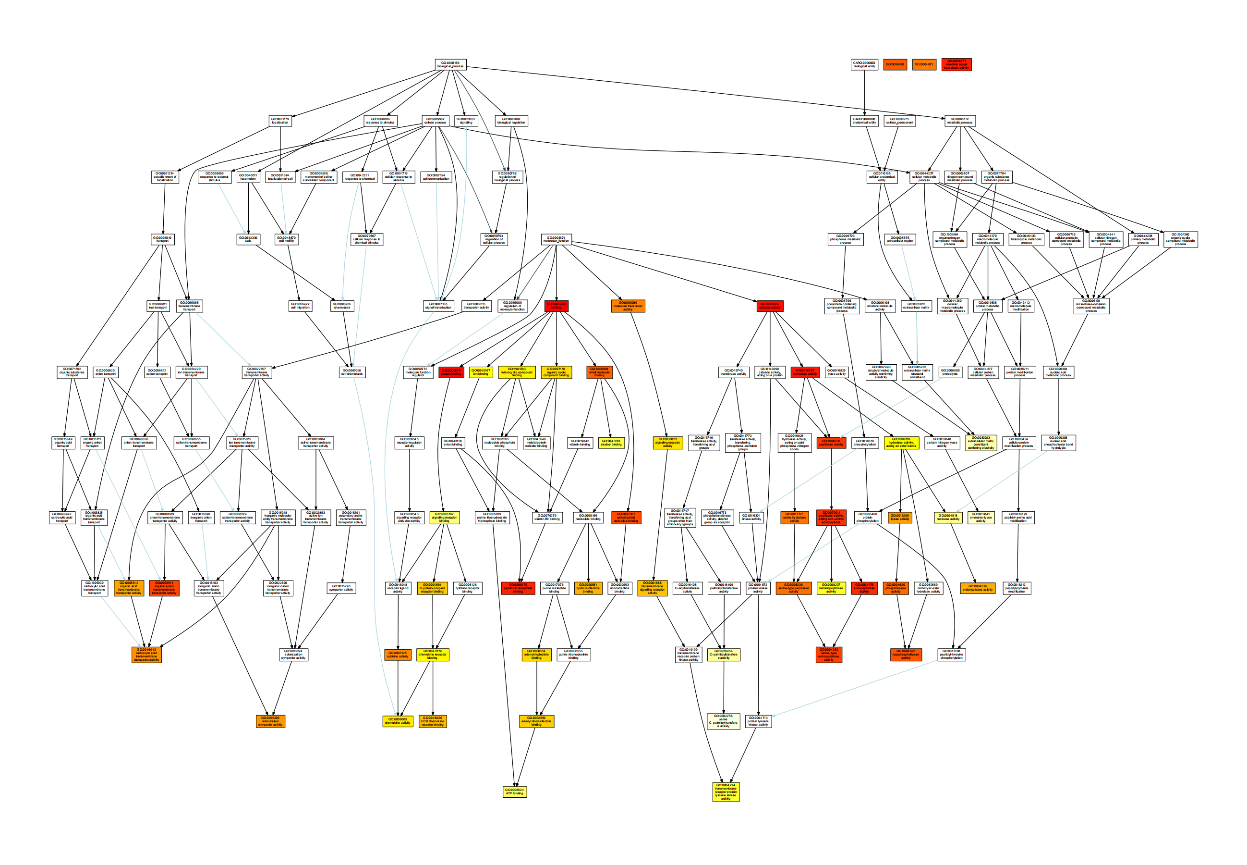  E |
| --- |
| 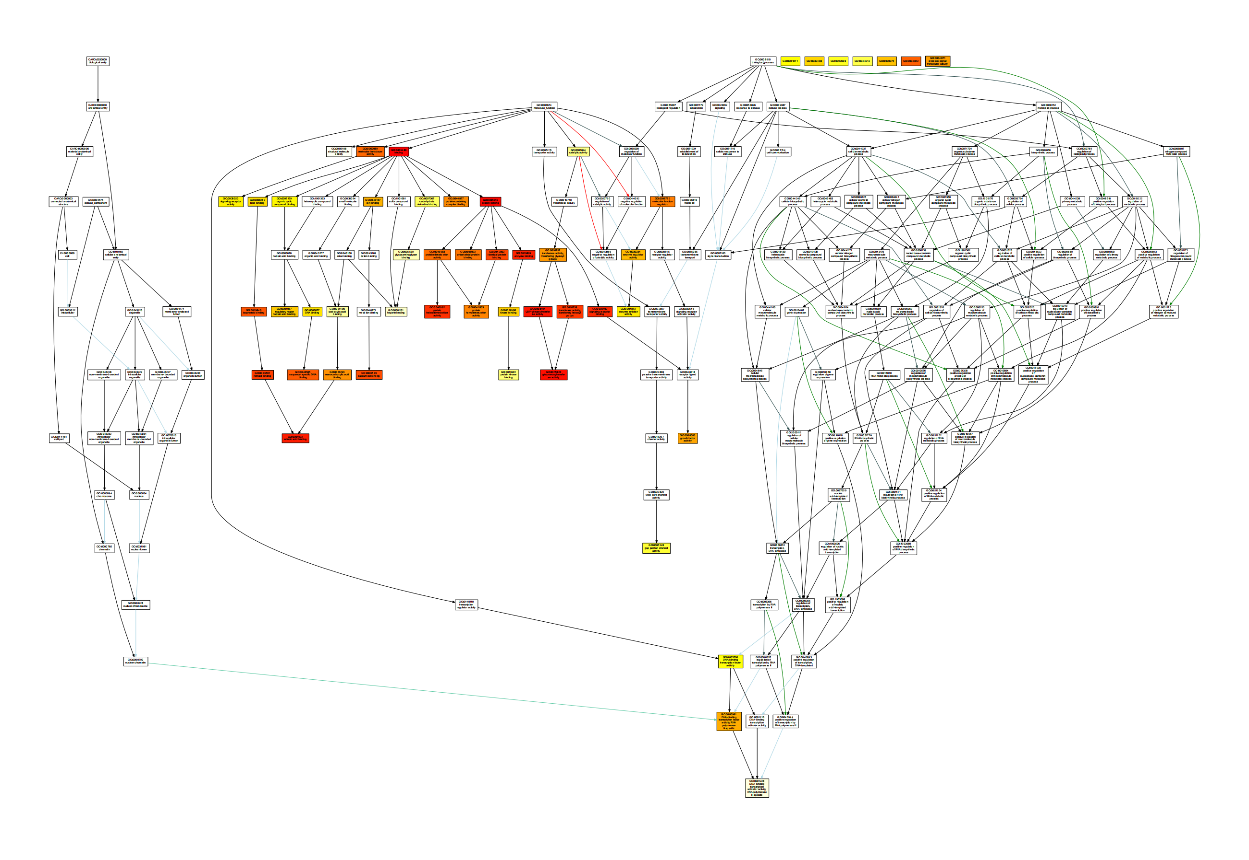  F |

**Figure S5. Results of gene ontology (GO) analysis**

A, GO analysis of upregulated RNAs; B, GO analysis of downregulated RNAs; C, involved biological processes of upregulated RNAs; D, involved biological processes of downregulated RNAs; E, involved biological functions of upregulated RNAs; F, involved biological functions of downregulated RNAs.

| 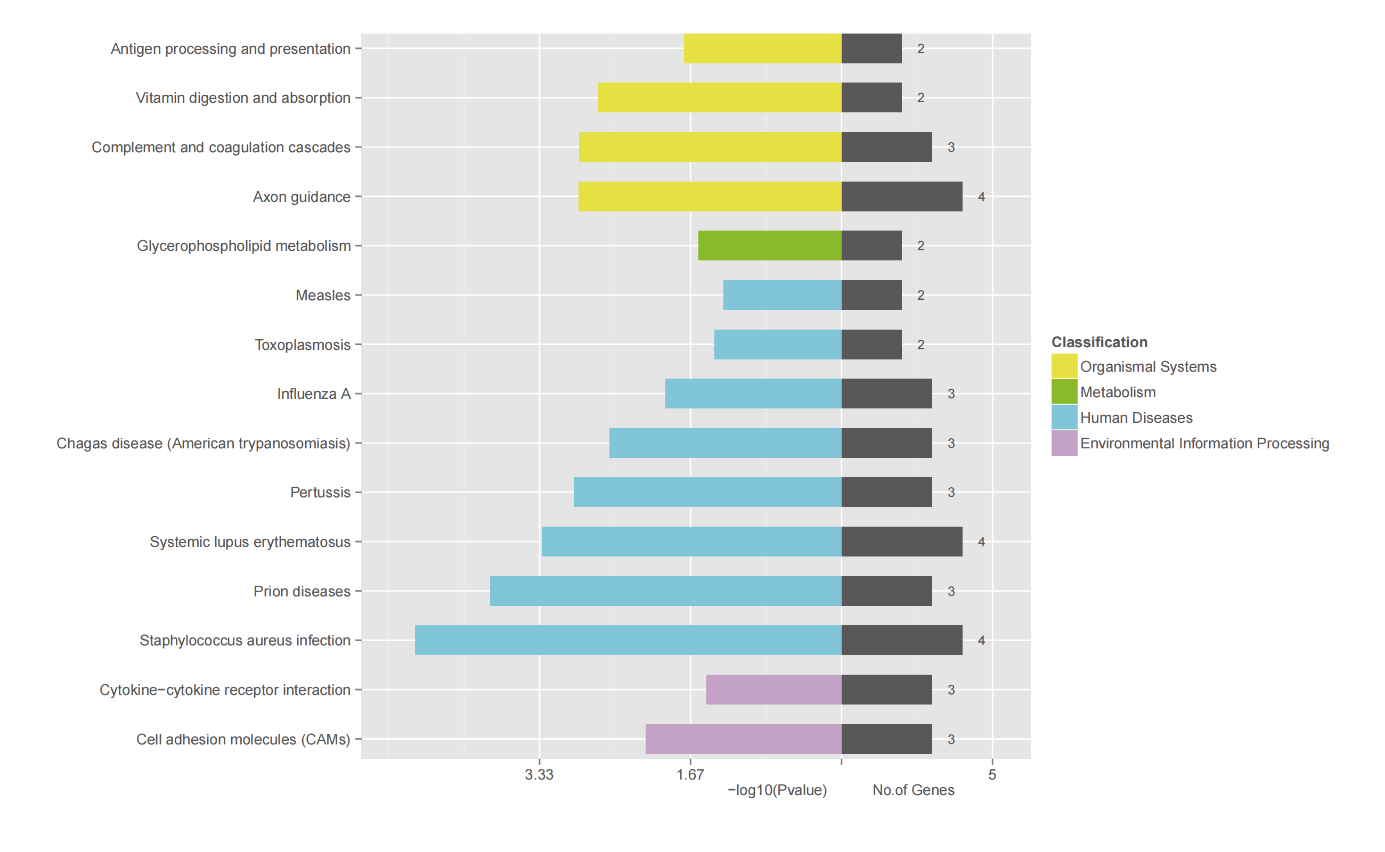  **A** |
| --- |
| 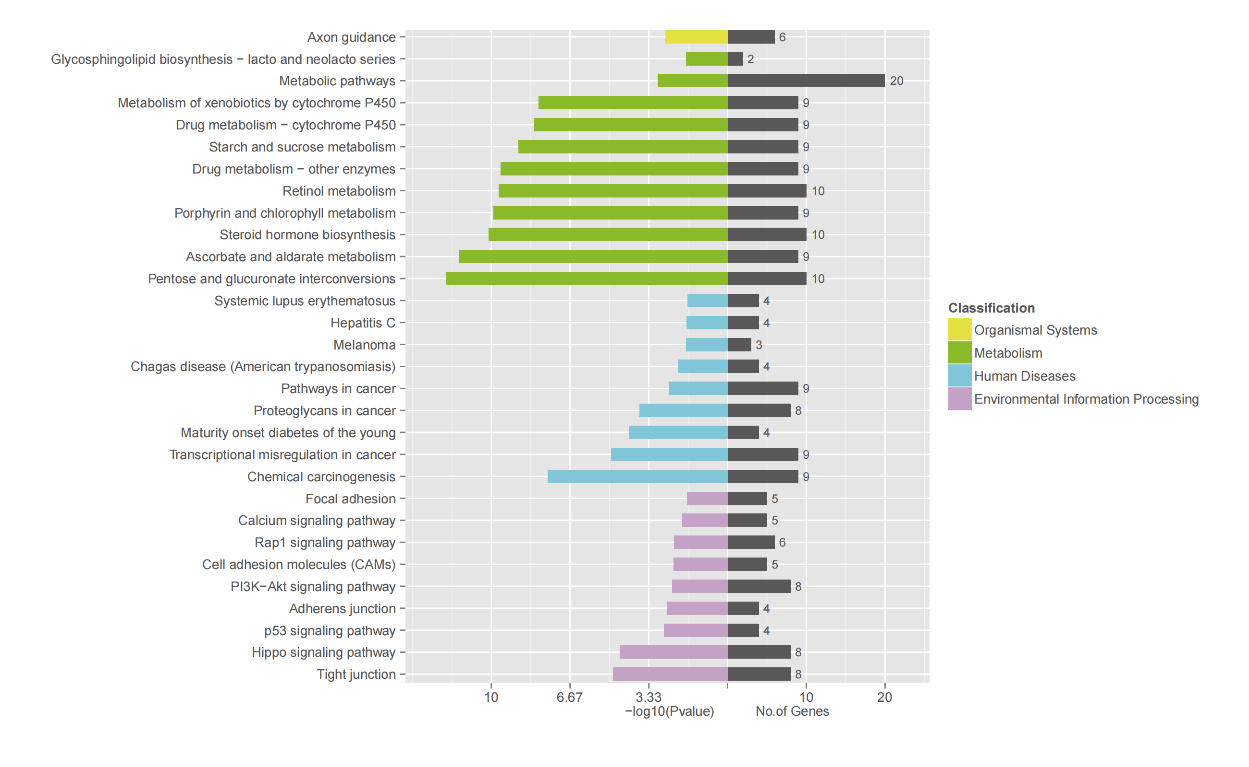  **B** |
| 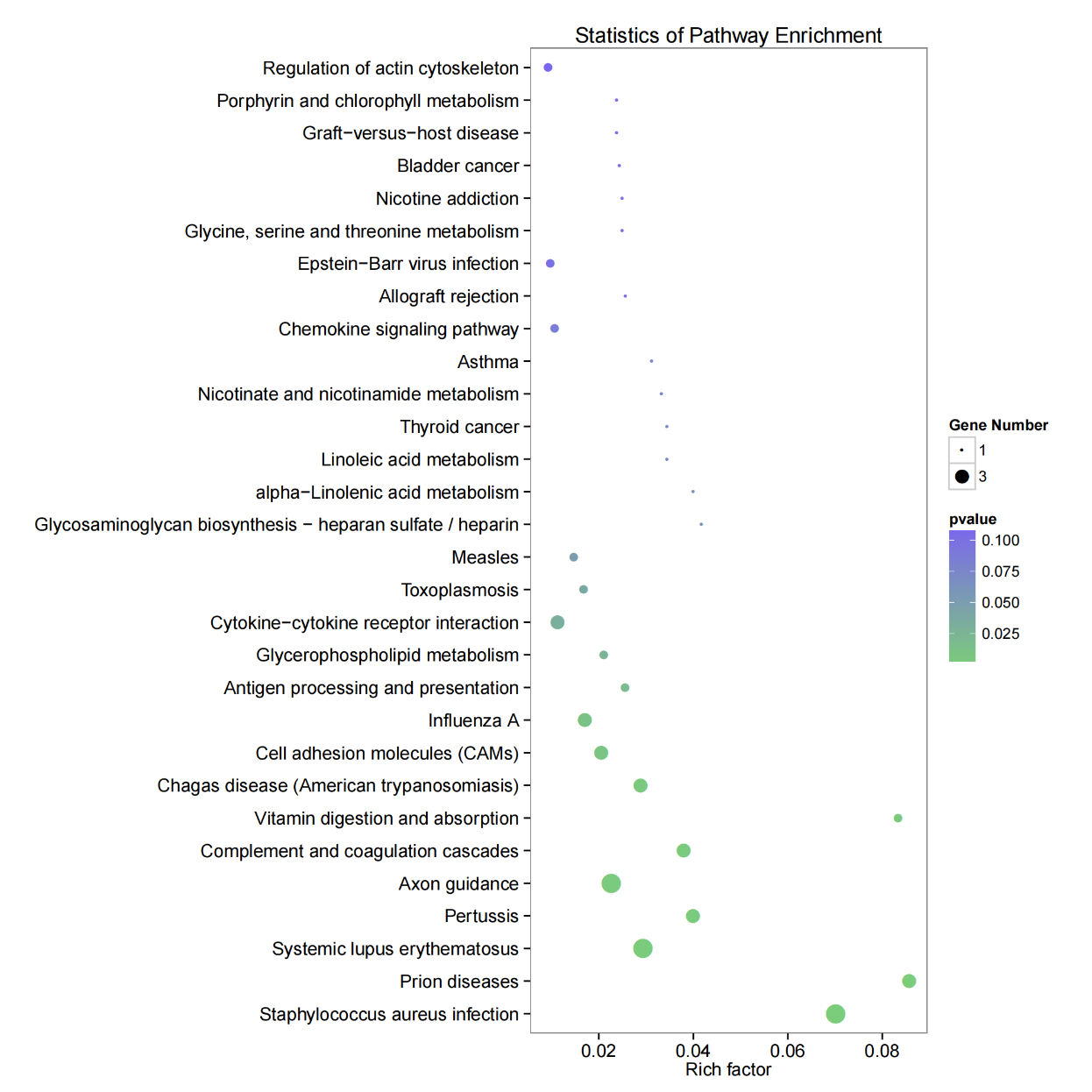  **C** |
| 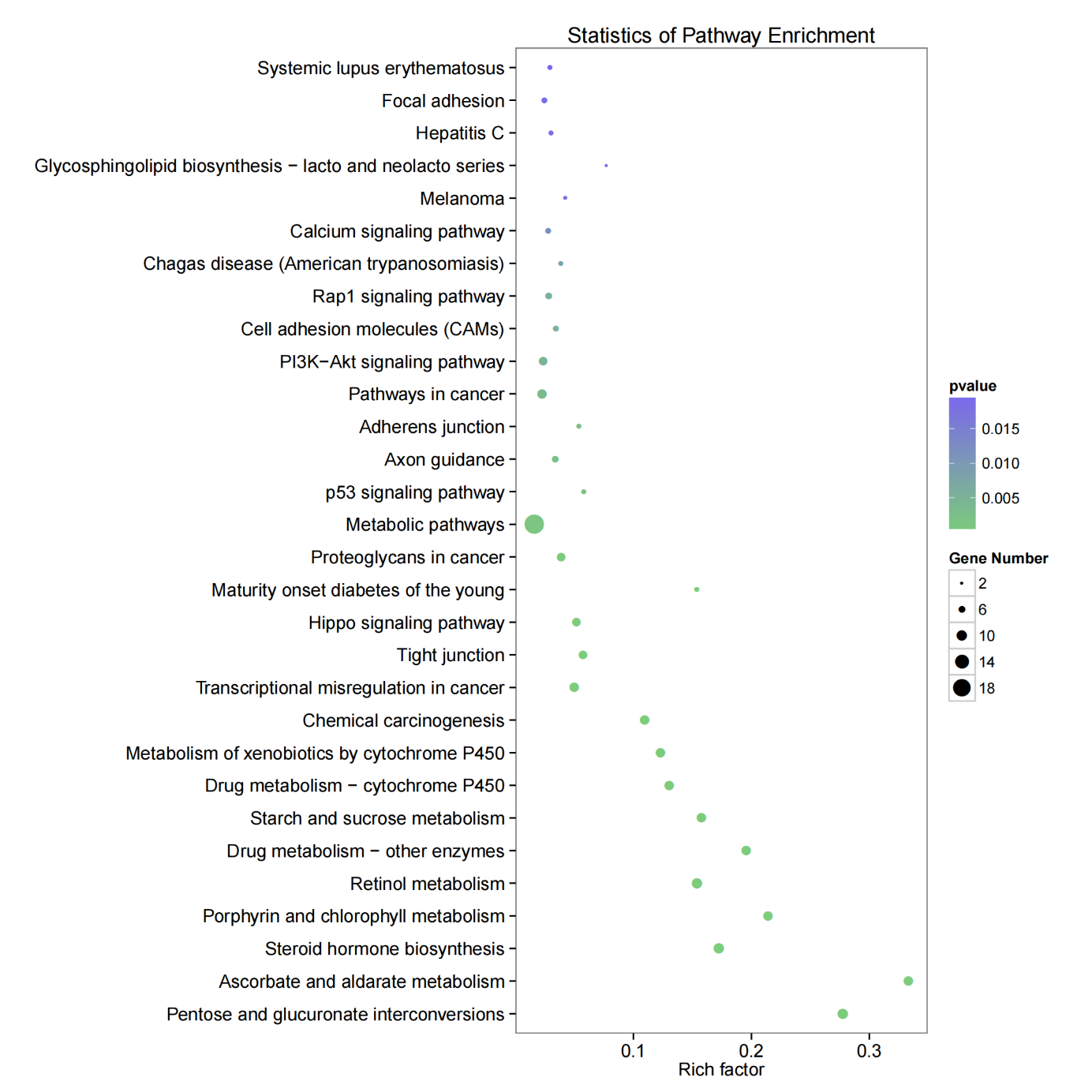  **D** |

**Figure S6. Results of Kyoto Encyclopedia of Genes and Genomes (KEGG) analysis**

A, KEGG of upregulated RNAs; B, KEGG of downregulated RNAs; C, KEGG points of upregulated RNAs; D, KEGG points of downregulated RNAs.

| 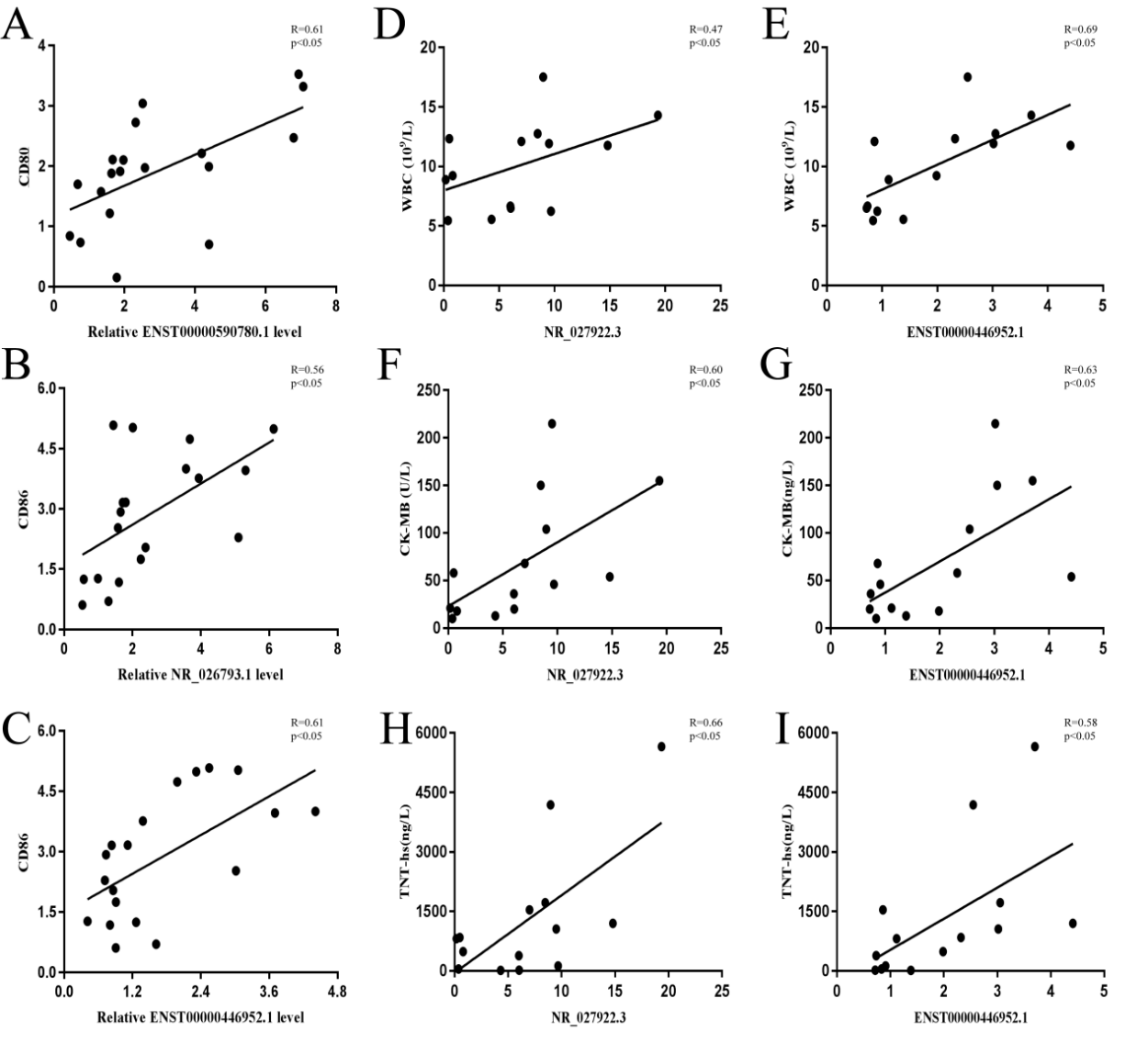 |
| --- |

**Figure S7. Pearson correlation coefficient of candidate lncRNAs and target Genes**

AMI: n=14; CON, n=5; *p<0.05, compared with CON.

LncRNA, long non-coding ribonucleic acid; AMI, acute myocardial infarction; CON, controls, healthy volunteers

| 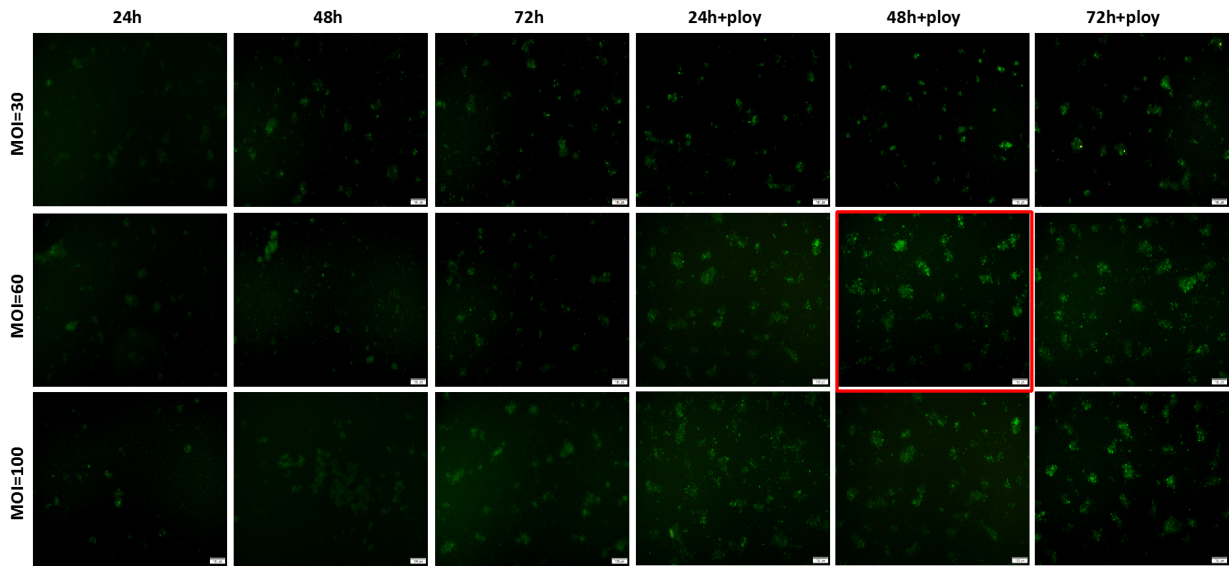 |
| --- |

**Figure S8. Fluorescence intensity of moDCs observed using an inverted fluorescence microscope**

Scale bar = 100 μm. moDC, monocyte-derived dendritic cell

| 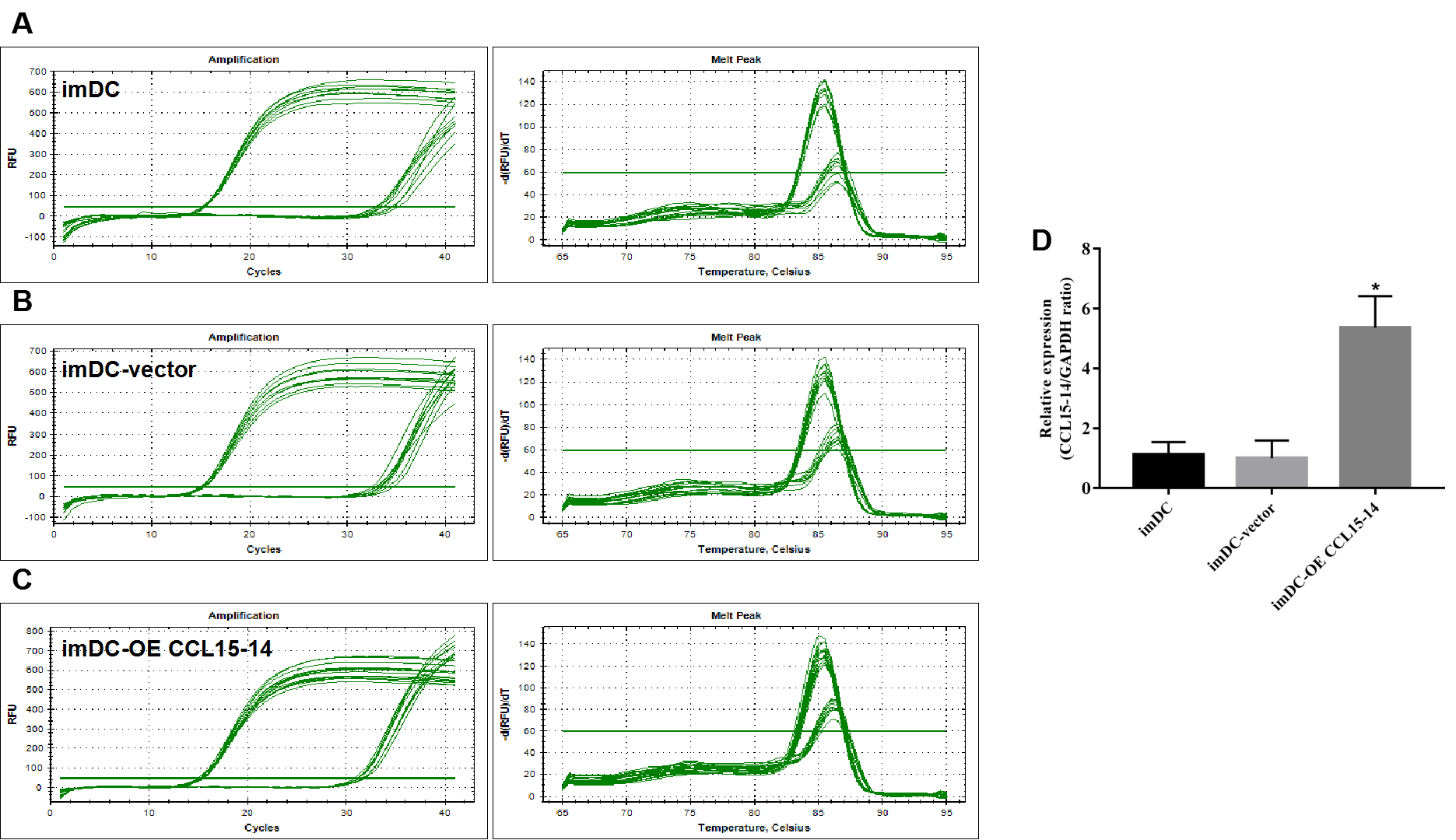 |
| --- |

**Figure S9. Reverse transcription-PCR outcomes: Amplification and dissolution curves and expression levels of CCL15-CCL14 after lentivirus over-expression**

C-C motif chemokine ligand (CCL); imDC, immature dendritic cells; imDC-vector, immature dendritic cell-vector; imDC-OE, immature dendritic cells overexpressing lentivirus; n=3 **p*<0.05, compared to the imDC group.

| 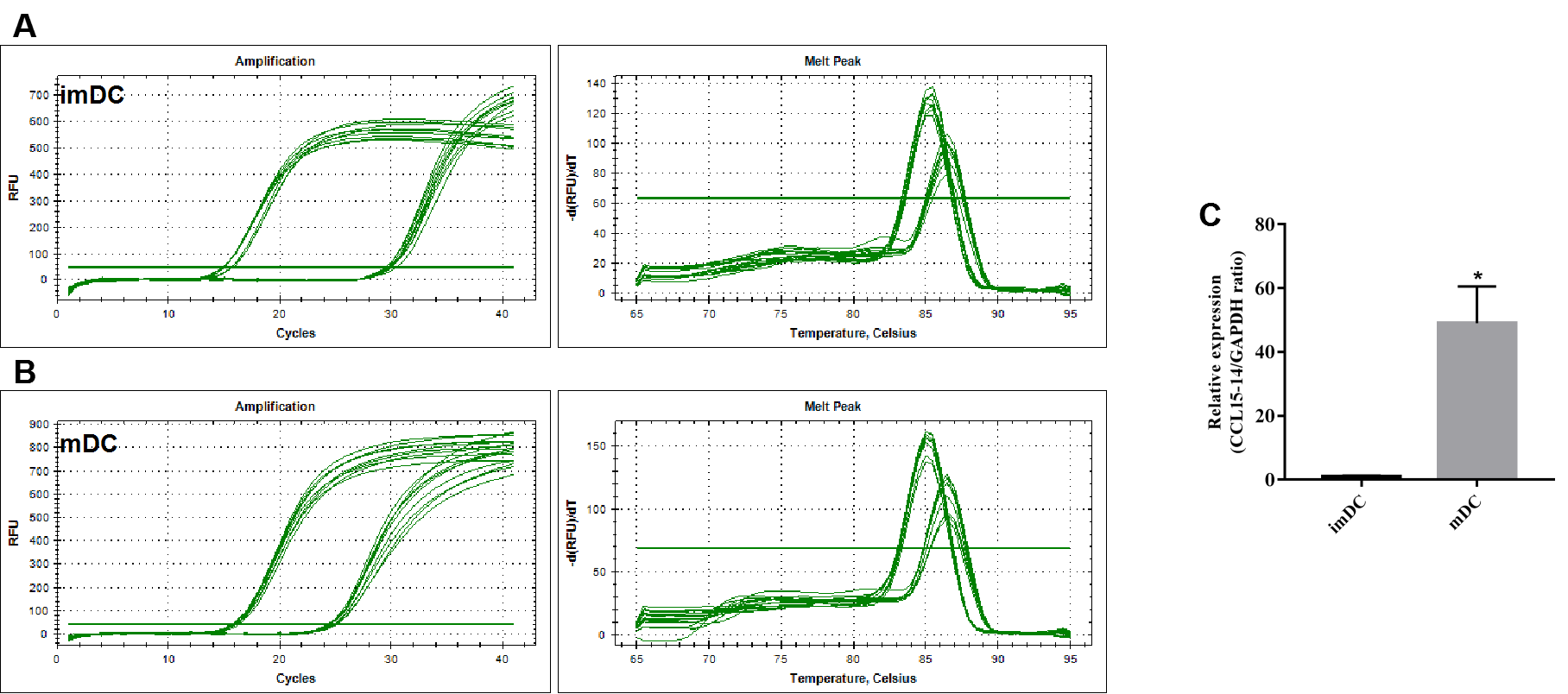 |
| --- |

**Figure S10. Reverse transcription-PCR results: amplification and dissolution curves and expression levels of CCL15-CCL14 after smart silencer-CCL15-CCL14**

C-C motif chemokine ligand (CCL); imDC, immature dendritic cells; mDC-vector, mature dendritic cells; n=3 **p*<0.05, compared to the imDC group.

| 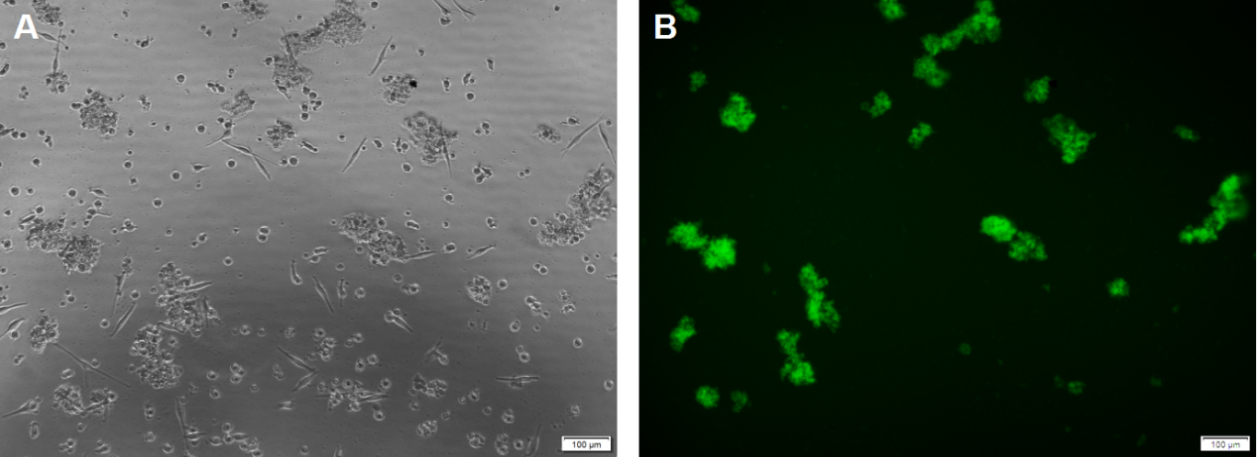 | 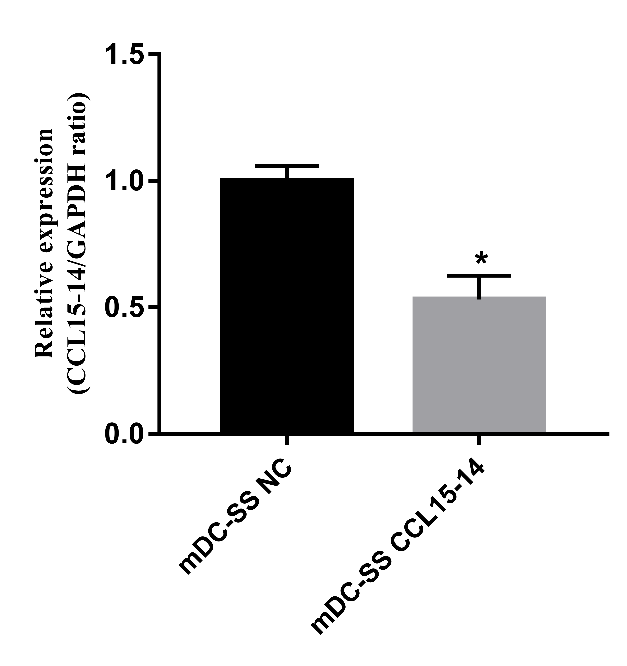  C |
| --- | --- |
| 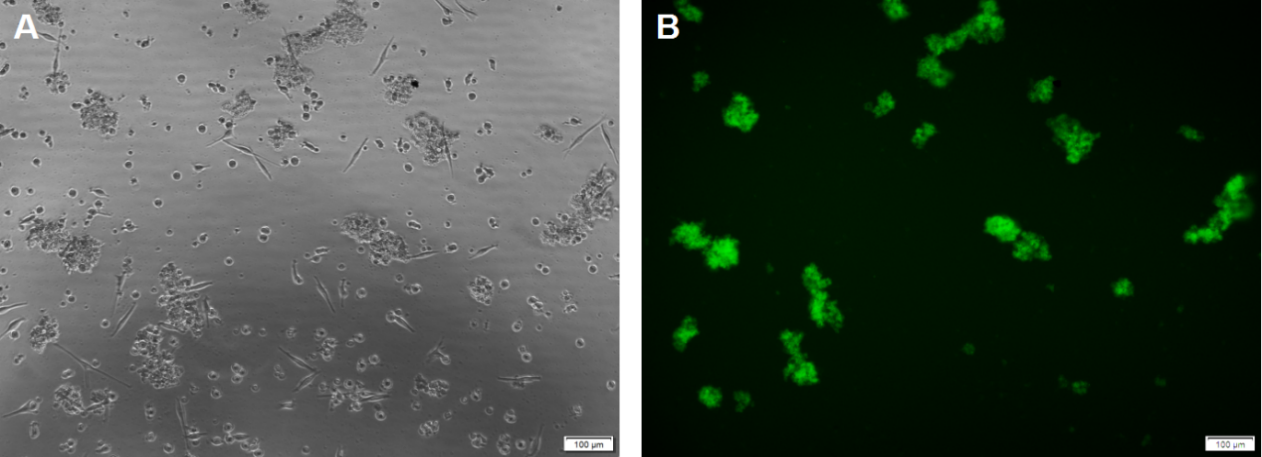 |

**Figure S11. The transfection effect of siRNA observed using an inverted fluorescence microscope and RT-PCR**

A and B, images of inverted fluorescence microscopy; C, results of reverse transcription-PCR; Scale bar =100 μm.

C-C motif chemokine ligand (CCL); GAPDH, glyceraldehyde-3-phosphate dehydrogenase; imDC, immature dendritic cells; mDC-vector, mature dendritic cells

| 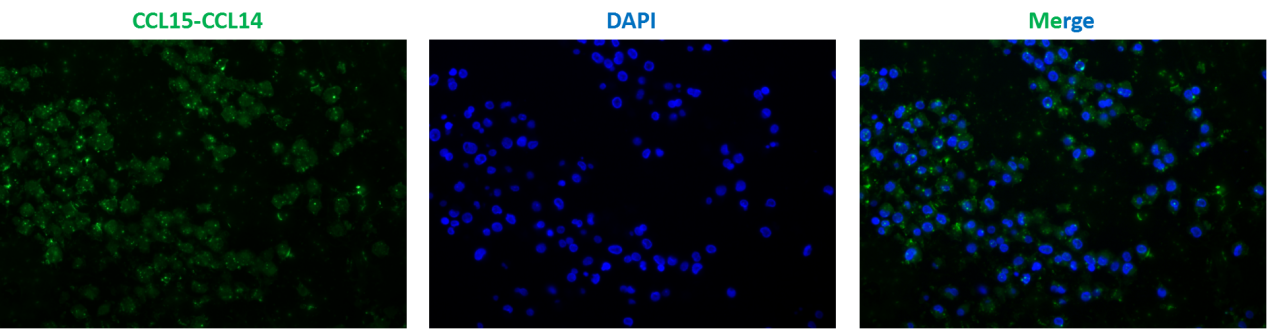 |
| --- |

**Figure S12. Immunofluorescence *in situ* hybridization of the subcellular localization of CCL15-CCL14**

Scale bar = 100 μm.

C-C motif chemokine ligand (CCL); DAPI,4',6-diamidino-2-phenylindole.
